# The genomic landscape of cutaneous SCC reveals drivers and a novel azathioprine associated mutational signature

**DOI:** 10.1038/s41467-018-06027-1

**Published:** 2018-09-10

**Authors:** Gareth J. Inman, Jun Wang, Ai Nagano, Ludmil B. Alexandrov, Karin J. Purdie, Richard G. Taylor, Victoria Sherwood, Jason Thomson, Sarah Hogan, Lindsay C. Spender, Andrew P. South, Michael Stratton, Claude Chelala, Catherine A. Harwood, Charlotte M. Proby, Irene M. Leigh

**Affiliations:** 1 Division of Cancer Research, Jacqui Wood Cancer Centre, School of Medicine, University of Dundee, Dundee, DD1 9SY UK; 2 Centre for Molecular Oncology, Barts Cancer Institute, Queen Mary University of London, London, EC1M 6BQ UK; 3 Department of Cellular and Molecular Medicine and Department of Bioengineering and Moores Cancer Center, University of California, San Diego, La Jolla, CA 92093 USA; 4 Centre for Cell Biology and Cutaneous Research, Barts and the London School of Medicine and Dentistry, Queen Mary University of London, London, E1 2AT UK; 5 Department of Dermatology and Cutaneous Biology, Thomas Jefferson University, Philadelphia, PA 19107 USA; 6 Cancer Genome Project, Wellcome Trust Sanger Institute, Hinxton, CB10 1SA UK

## Abstract

Cutaneous squamous cell carcinoma (cSCC) has a high tumour mutational burden (50 mutations per megabase DNA pair). Here, we combine whole-exome analyses from 40 primary cSCC tumours, comprising 20 well-differentiated and 20 moderately/poorly differentiated tumours, with accompanying clinical data from a longitudinal study of immunosuppressed and immunocompetent patients and integrate this analysis with independent gene expression studies. We identify commonly mutated genes, copy number changes and altered pathways and processes. Comparisons with tumour differentiation status suggest events which may drive disease progression. Mutational signature analysis reveals the presence of a novel signature (signature 32), whose incidence correlates with chronic exposure to the immunosuppressive drug azathioprine. Characterisation of a panel of 15 cSCC tumour-derived cell lines reveals that they accurately reflect the mutational signatures and genomic alterations of primary tumours and provide a valuable resource for the validation of tumour drivers and therapeutic targets.

## Abstract (web summary)

It is known cutaneous squamous cell carcinoma (cSCC) involves a high tumour mutation burden. Here the authors identify common cSCC mutated genes, copy number changes, altered pathways and report the presence of a novel mutation signature associated with chronic exposure to the immunosuppressive drug azathioprine.

## Introduction

The incidence of keratinocyte skin cancers in fair skinned populations currently exceeds that of all other cancers combined and is increasing year on year in our ageing population. More than one million new cases of cutaneous squamous cell carcinoma (cSCC) are diagnosed annually in the US, with significant health economic implications^1^. In contrast to most other epithelial malignancies, more than a third of patients develop multiple primary cSCC. This is especially true in immunosuppressed individuals with evidence in organ transplant recipients (OTR) of a more than 100-fold increased risk of developing cSCC^2^. Although many cSCC are effectively treated with surgery or radiotherapy, morbidity is significant, particularly as tumours frequently occur on cosmetically sensitive sites. Metastasis occurs in ~5% of cases and cSCC is responsible for approximately one-quarter of skin cancer-related deaths and estimates for annual mortality are similar to those for melanoma in parts of the USA^3^. There are few effective treatments for advanced cSCC, with five-year survival of less than 30% reported for metastatic disease^4^.

Cutaneous SCC is poorly understood at a molecular level and the drivers of disease progression have not been fully elucidated. Whole-exome sequencing (WES) has revealed a high mutation rate with on average 50 mutations per megabase pair DNA^5^. Additionally, gross chromosomal aberrations and numerous copy number alterations (CNA) indicative of genetic instability create a complex molecular landscape. Epidemiological and clinical evidence supports cumulative life time exposure to ultraviolet radiation (UVR) as the principle environmental carcinogen responsible for cSCC and the majority of mutations found in cSCC contain a ‘UV signature’. Our understanding of the genomic landscape of cSCC is further complicated by keratinocyte clones in apparently normal sun-exposed skin^6^ bearing mutations in cSCC tumour suppressor genes (TSGs) such as *NOTCH1/2* and *TP53*^5,7–10^. There is evidence that the mutational profile of cSCC harbours similarities to those of other epithelial tumours, including head and neck, lung and oesophageal SCCs (reviewed in ref. ^11^) and the squamous subtype of pancreatic SCC^12^, but comparisons are limited by small cSCC data sets.

Analyses of the patterns of mutations in cancer genomes has revealed the presence of distinct ‘mutational signatures’^13,14^. They are described using a simple classification based on the six classes of single base mutations in combination with the base 5' and 3' to each mutation and characteristic UVR-induced C > T mutations at pyrimidines appear to predominate in all cSCC studies to date (COSMIC signature 7).

Here, we perform WES of 40 primary cSCC from both immunosuppressed (IS) and immunocompetent (IC) patients classified according to histological differentiation status (well-differentiated vs. moderately/poorly differentiated categories). We have taken an integrated bioinformatics approach with the intention of identifying a limited number of actionable pathways onto which this multiplicity of genetic events will converge. In addition to mutational, chromosomal and clonality analyses, a mutational signature analysis has been performed, revealing a novel mutational signature associated with patients who have been exposed to the immunosuppressive agent, azathioprine.

Furthermore, we describe the molecular characterisation of 15 primary cSCC cell lines. These reflect the mutational signatures, mutational and genomic alteration profiles and gene expression changes of primary tumours and provide a resource for investigating the contribution of genes, pathways, and processes for cSCC progression.

## Results

### Mutational burden and signatures

**Fig. 1:**
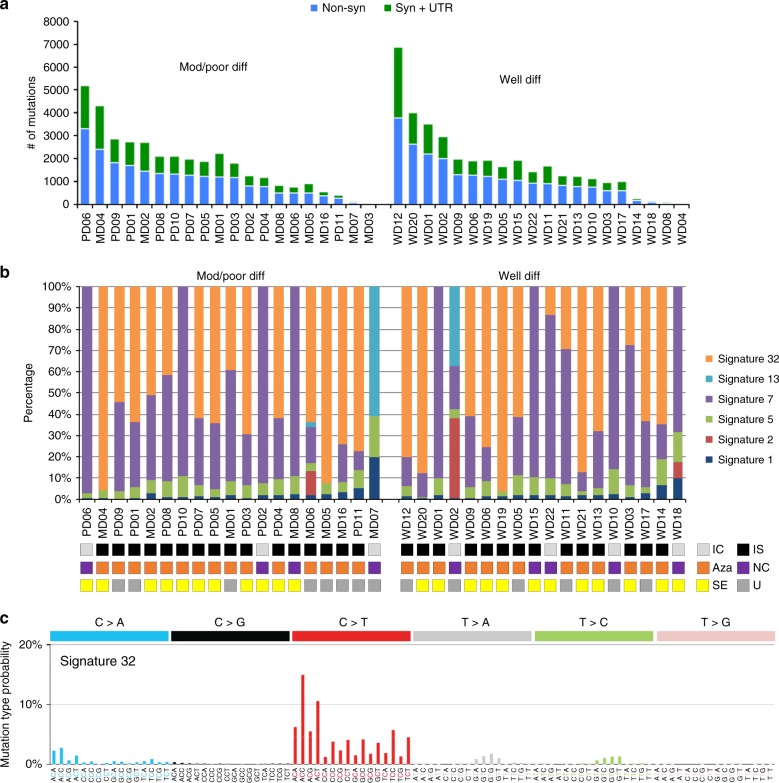
Number of somatic mutations and mutation signatures across 40 cSCC samples in the moderate/poor and well-differentiated groups. **a** Number of nonsynonymous, synonymous and UTR mutations across 40 samples. **b** Mutation signature compositions across 37 of the 40 samples with sufficient mutation depth, in terms of moderate/poor and well-differentiated groups. IC immunocompetent, IS immunosuppressed, Aza confirmed azathioprine exposure, NC no confirmed azathioprine exposure, SE sun exposed site, U unknown if sun exposed site. **c** A novel signature, termed signature 32, predominately C > T mutations (75%) in combination with C > A, T > A, and T > C mutations, was identified as one of the dominant signatures. This signature is putatively associated with azathioprine treatment

Forty cSCC samples from 37 patients (Supplementary Data 1) were analysed/reanalysed by WES (including 30 previously described)^5,15^. Twenty-nine patients were organ transplant recipients receiving immunosuppressive drugs and one was immunosuppressed for Crohn’s disease. This sample set contained equal numbers of well-differentiated (WD) vs. poorly/moderately differentiated (MD/PD) tumours (20 samples each). WES coverage averaged 54.3 × (range 25.8–79.1×) with 91% of bases covered > 10 × (range 73.5–97.8%) and 81.4% > 20 × (range 47.2**–**93%) (Supplementary Data 2). We identified 71,242 somatic mutations in the tumours (Supplementary Data 3). The number of nonsynonymous and synonymous mutations varied across each group averaging 1795 somatic variants in WD (range 23**–**6867) and 1793 somatic variants in MD/PD (range 13**–**5188) (Fig. 1a and Supplementary Data 3). Computational analysis of patterns of somatic mutations^13^ has enabled the identification of 30 distinct mutational signatures from more than 12,000 samples from a wide range of cancer types^14,16,17^ (for a complete set of currently known signatures see http://cancer.sanger.ac.uk/cosmic/signatures). Many of these signatures have been attributed to environmental exposure to mutagens^14,16–18^. Mutational signature analysis was performed on 37/40 of the cSCC whole-exome sequences (Fig. 1b and Supplementary Data 4). Signature 7 was observed in 33 samples (Fig. 1b), which reflected the C > T mutations of dipyrimidines and has previously been described as a ‘UVR signature’^14^ (Supplementary Fig. 1B). Twenty-one of these UVR signature positive tumours were from confirmed sun exposed sites (Fig. 1b; head and neck, wrist and hand, Supplementary Data 1) and the remaining 12 were from sites which included the leg, chest, shoulder and back, which may also have been sun exposed (Supplementary Data 1). MD07, which displays no evidence of UVR exposure, was isolated from the foot of the patient and was, therefore, unlikely to be sun exposed (Fig. 1b and Supplementary Data 1, 4).

A previously undescribed mutational signature, termed signature 32 here-after, was found in 27 samples from both the WD and MD/PD tumours (Fig. 1b, orange bars). It is predominately C > T mutations (75%) in combination with C > A, T > A, and T > C mutations (Fig. 1c). There seems to be a clear presence for C > X at ApCpN and T > X at GpTpN. This novel mutational signature exhibits a very strong transcriptional strand bias potentially indicating an interplay with transcription coupled nucleotide excision repair (TC-NER) due to adducts on guanine and adenine (Supplementary Fig. 1A). The pattern of signature 32 is distinct from any of the previously known COSMIC mutational signatures, including signatures that have a pattern of mutations with predominately C > T transitions such as signature 7. Only two of the COSMIC mutational signatures exhibit a correlation > 0.5 between their respective patterns and that of signature 32. The pattern of signature 30 (attributed to mutations in the base excision repair gene NTHL1^19^) has a correlation of 0.58 with signature 32 and that of signature 11 (attributed to exposure to alkylating agents^13,14^) has a correlation of 0.65 with signature 32. These correlations are lower than those of other distinct COSMIC signatures, for example COSMIC signatures 1 and 6 have a correlation of 0.81^13,14^. Further, signatures 11 and 30 do not exhibit the same transcriptional strand bias as signature 32. Thus, signature 32 is an entirely novel mutational signature.

Signature 32 was prominent in many of the samples from immunosuppressed patients (Fig. 1b) indicating a potential association with exposure to immunosuppressant drugs. Most immunosuppressed patients received azathioprine (Fig. 1b) although this was usually part of a complex regimen involving at least one other immunosuppressive drug (Supplementary Data 1). However, three of the tumours with signature 32 were from patients who had received azathioprine alone (MD02, PD01, WD03) and both tumours analysed from immunosuppressed patients whom had not received azathioprine (MD08, WD15) did not exhibit mutational signature 32 (Fig. 1b and Supplementary Data 1, 4). There is a significant association only between a confirmed history of azathioprine exposure and the presence of signature 32 (Fisher’s exact test, *p* < 0.0001; Supplementary Fig. 2a) and not exposure to the other immunosuppressant drugs (Supplementary Fig. 2a). We did not identify any other significant associations between any of the mutational signatures and exposure to any of the immunosuppressant drugs (Supplementary Fig. 2b). Furthermore, analysis of treatment times revealed a strong positive correlation with the estimated time of azathioprine exposure and the prevalence of signature 32 (Spearman’s rank order correlation *r*_s_(26) = 0.679, *p* < 0.0001, Supplementary Fig. 3). Taken together these findings indicate that chronic exposure to azathioprine correlates with the presence of mutational signature 32. All tumours contained lower levels of signatures 1 and 5, which are found in most cancers and are attributed to so called ‘clock-like’ mutational processes^20^ (Fig. 1b and Supplementary Data 4). Four tumour samples also contained mutational signature contributions from Signatures 2 and 13 (MD06, MD07, WD02 and WD18; Fig. 1b and Supplementary Data 4), which are attributed to the activity of the AID/APOBEC family of cytidine deaminases converting cytosine to uracil^14^.

### Somatic copy number aberrations

**Fig. 2:**
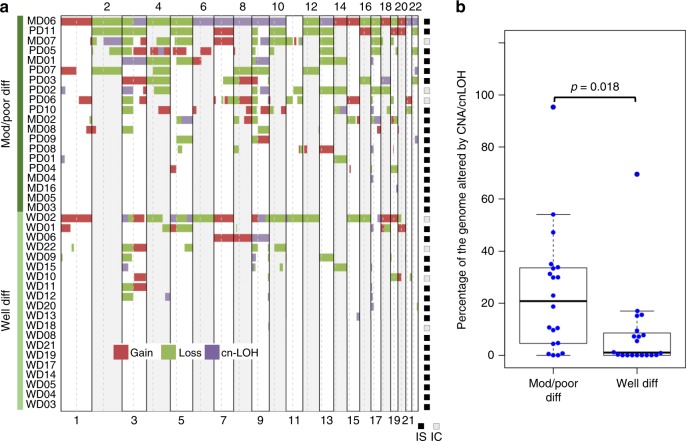
Somatic copy number aberrations (SCNA) and LOH events in 40 cSCC samples. **a** OncoPrint of copy gain, loss and copy-neutral (CN)-LOH segments across moderate/poor and well-differentiated groups. IS immunosuppressed, IC immunocompetent. **b** Box-and whisker plot indicating the percentage of the genome altered by CNA/LOH across the two groups. Individual values were shown by the blue dots. Moderate/poor differentiated group had significantly higher percentage of altered genome than well-differentiated group (Student’s *t*-test, two sided, *p* = 0.018)

Somatic copy number aberrations (CNA) and loss of heterozygosity (LOH) events were identified across WD and MD/PD samples (Supplementary Data 5), with MD/PD samples having more large-scale genomic events than WD samples (Fig. 2a, b). 62.5% of the samples (25/40) had detectable copy number variations (CNVs) in at least 2 regions, which is consistent with previous observations^21–24^ with a significant positive correlation with previous studies for top frequent CNAs (Supplementary Fig. 4, Pearson’s correlation *r* = 0.37, *p* = 0.015). GISTIC^25^ analysis further identified significantly amplified and deleted regions (*q* < 0.1, Supplementary Data 6), including amplifications at 3q26, 5q22, 7q21, 11q22 and 15q11, and deletions at 3p12, 5q13 and 9q21. Amplifications at 3q^21–24^, 5q^21^, 7q^24^, and copy losses at 3p^21–24^ and 5q^24^ have also previously reported. Frequent gain of 9q and loss of 9p are also observed in this and previous studies of cSCC (Supplementary Data 7).

### Significantly mutated genes (SMGs) in cSCC

**Fig. 3:**
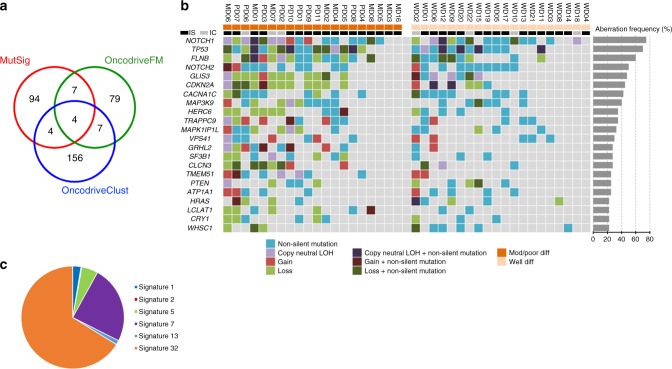
Twenty-two SMGs identified in 40 cSCC samples. **a** Venn diagram of overlap of significantly mutated genes as assessed by MutsigCV, OncodriveFM and OncodriveClust. **b** Mutation OncoPrint of the 22 SMGs identified by at least two of the three methods, with their aberrational frequencies also indicated with percentage bars. IS immunosuppressed, IC immunocompetent. **c** Pie chart of the mutational signature contribution to SMG mutations

We employed three statistical mutational significance methods; MutsigCV^26^ (significance cutoff *p* < 0.05, Supplementary Data 8), Oncodrive-FM^27^ (significance cutoff *q* < 0.05, Supplementary Data 9) and Oncodrive-CLUST^28^ (significance cutoff *q* < 0.05, Supplementary Data 10). These identified 109, 97 and 171 SMGs respectively totalling 351 unique genes (Fig. 3a and Supplementary Data 8–10). We identified 22 SMGs by at least two methods (Fig. 3a, b). This revealed recurrent mutations in a number of genes previously implicated in human cSCC including *NOTCH1/2*, *TP53* and *CDKN2A* (Fig. 3b and Supplementary Data 11, 12, Supplementary Fig. 5). The frequency and statistical significance of these potential driver genes are consistent with previous exome^10^ and targeted sequencing studies^9^ (Supplementary Data 13).

Additional SMGs included *HRAS*, *MAP3K9*, *PTEN*, *SF3B1*, *VPS41* and *WHSC1* (Fig. 3b, Supplementary Fig. 6 and Supplementary Data 14) all of which have known genetic alterations in human malignancies^29–38^. We identified oncogenic activating mutations in *HRAS* in three tumours (Fig. 3b and Supplementary Fig. 6), which have previously been identified in 3**–**20% of cSCC^9,10^ (Supplementary Data 13) and keratoacanthomas (reviewed in ref. ^4^). Of note, 10% of the samples exhibit copy number loss of *HRAS* (Fig. 3b), which has been observed by others^9^, warranting a need for better understanding of the role of HRAS in cSCC. *PTEN* alterations have been previously described in cSCC^9^ (Supplementary Data 13), but the other SMGs including *FLNB*, *GLIS3*, *CACNA1C*, *HERC6*, *TRAPPC9*, *MAPK1P1L* (Fig. 3b, Supplementary Fig. 7 and Supplementary Data 15), *GRHL2*, *CLCN3*, *TMEM51*, *ATP1A1*, *LCLAT1* and *CRY1* (Fig. 3b, Supplementary Fig. 8 and Supplementary Data 16) are of unknown significance in cancer and present with no obvious hotspot mutations. Non-silent mutations in a few of these SMGs have also been observed in cSCC^10^ (Supplementary Data 13). Comparison of the frequency of alterations in the 22 SMGs between tumours isolated from immunosuppressed and immunocompetent patients revealed no significant difference in mutation frequencies indicating that tumours that arise in these patient groups have common drivers (Fig. 3b and Supplementary Data 17). Next, we assessed the probability for each mutational somatic substitution in the 22 SMGs to be caused by each of the operative mutational signatures. Overall the majority of the SMG gene mutations are caused by azathioprine signature 32 (66.2%): 24.4% are due to UVR driven signature 7; 7.8% are caused by clock-like mutational signatures^20^ (signatures 1 and 5) and the remaining 1.7% by APOBEC related signatures 2 and 13, suggesting the driving mutational processes responsible for the initiation and progression of the tumours in our sample set are due to a combination of UVR and azathioprine exposure (Fig. 3c and Supplementary Data 18).

### Inference of clonal evolution and order of genetic changes

**Fig. 4:**
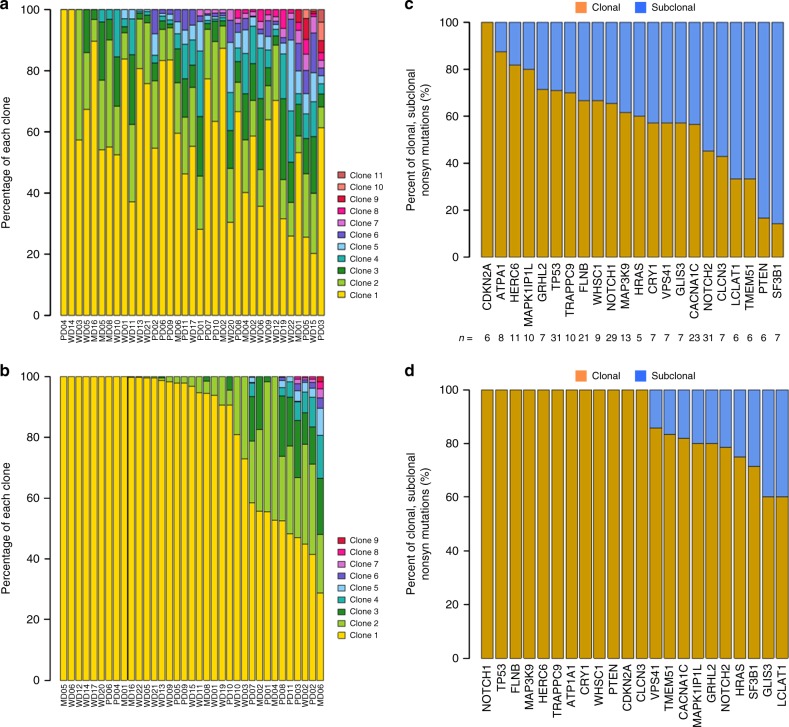
Clonality analysis of cSCC tumour samples. Clonality analysis of 35 cSCC exomes using EXPANDS (**a**) and SciClone (**b**). Clonality analysis of 22 SMGs identifying clonal and subclonal nonsynonymous mutations using EXPANDS (**c**) and SciClone (**d**). The number of nonsynonymous mutations in each gene was also shown

Next, we performed clonal analysis, integrating somatic mutations (SNVs and indels) and CNAs using EXPANDs^39^, and SciClone^40^ which have been used to infer patterns of clonal evolution^41,42^. In 35 of 40 cSCC, WES data were sufficient for the identification of dominant clone and nested subpopulations in each sample, thus allowing for the differentiation between clonal and subclonal mutations. EXPANDS and SciClone had concordant estimates of tumour purity (Supplementary Fig. 9, Pearson’s correlation *r* = 0.9, *p* = 3.42e-13, Supplementary Data 19). There was no correlation between the number of somatic mutations identified in our samples and tumour purity (Pearson’s correlation *r* = 0.10, *p* = 0.57). Clonality analysis showed tumour heterogeneity across our samples, varying from 1 to 11 clones as estimated by EXPANDS (Fig. 4a) and 1 to 9 clones as estimated by SciClone (Fig. 4b) with some correlation in the number of clusters estimated by the two methods (Supplementary Fig. 10). 26/35 (74%) had at least two clones, 10/35 (29%) had at least three clones and 7/35 (20%) had at least five clones identified by both programmes (Fig. 4a, b and Supplementary Data 20) indicating a high degree of heterogeneity in cSCC.

For the 22 SMGs, we assessed the order of driver mutation acquisition inferred from the aggregate frequencies at which they were found to be clonal or subclonal^43^. We characterised the clonal and subclonal features of the nonsynonymous mutations for these 22 genes with 74% of all clonal mutations called by EXPANDS also identified as clonal by SciClone (Supplementary Fig. 10). Over 70% of mutations in *CDKN2A*, *ATP1A1*, *HERC6*, *MAPK11P1L*, *GRHL2* and *TP53* were clonal using both methods implying alterations in these genes tended to arise earlier during cSCC development (Fig. 4c, d). Rank order of clonality similarly indicated that mutations in these genes and also those in *NOTCH1*, *TRAPPC9*, *FLNB* and *MAP3K9* may also be early events, whereas mutations in *HRAS*, *VPS41*, *GLIS3*, *CACNA1C*, *NOTCH2*, *CLCN3*, *LCLAT1*, *TMEM51* and *SF3B1* appeared to accumulate a higher proportion of subclonal nonsynonymous mutations (Fig. 4c, d and Supplementary Data 21), indicating that lesions in these genes are more likely to occur later.

### Integration of genomic drivers and gene expression profiles

**Fig. 5:**
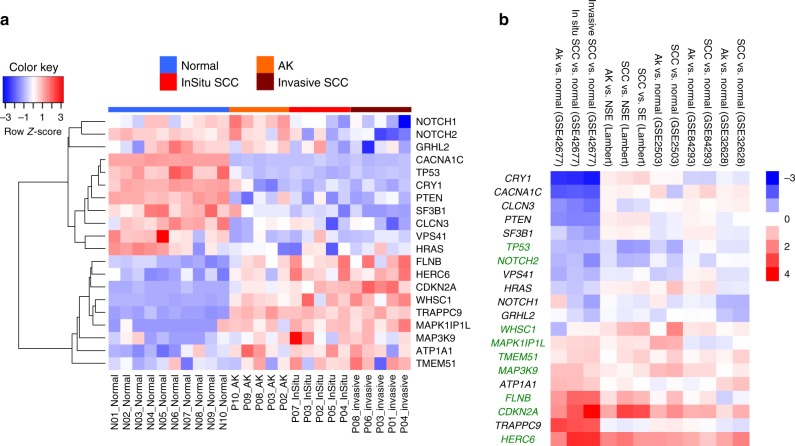
Expression profiles of driver genes in five independent gene expression data sets. **a** Expression heatmap of 20 SMGs that were also expressed across normal, AK, in situ and invasive SCC samples from GSE42677. **b** Log_2_ fold changes (FC) of AK vs. normal and SCC vs. normal of 20 SMGs from GSE42677, Data set 1 (Lambert), GSE2503, GSE84293 and GSE32628. Normal normal skin, NSE non-sun exposed normal skin, SE Sun exposed skin, AK actinic keratosis. Significant genes in 3 out of 5 data sets were highlighted in green. Within the heatmap, red colour indicates the upregulation in AK or SCC compared to normal control, which blue colour indicates the downregulation in AK or SCC in relation to normal skin

To investigate potential tumour suppressor or tumour promoter roles of our 22 SMGs and genes encoded within areas of significant CNAs, we analysed gene expression profiles (GEP) in five independent data sets comprising samples from normal skin, actinic keratoses (AK) and SCC. These included GSE45216^44^ coupled with additional samples described here as Data set 1 (Supplementary Data 22), GSE42677^45^, GSE2503^46^, GSE84293^7^ and GSE32628^21^. *GLIS3* and *LCLAT1* were determined to have a low level of expression in normal and/or lesional skin across samples and were excluded from this analysis (see Methods). The remaining 20 SMGs were clearly separated into two clusters in GSE42677 (Fig. 5a). One cluster was highly expressed in normal skin and became downregulated in AK and SCC, and the other cluster demonstrated the opposite trend (Fig. 5a). The down- and upregulated patterns for these genes were largely consistent between the five data sets (Fig. 5b). Among them, *TP53* and *NOTCH2* were downregulated in SCC compared to normal skin, while seven genes *HERC6*, *CDKN2A*, *FLNB*, *MAP3K9*, *TMEM51*, *MAPK1IP1L* and *WHSC1* were upregulated in SCC relative to normal skin in at least 3 out of 5 data sets (Fig. 5b) suggesting respective potential tumour suppressing and tumour promoting roles in cSCC development.

**Fig. 6:**
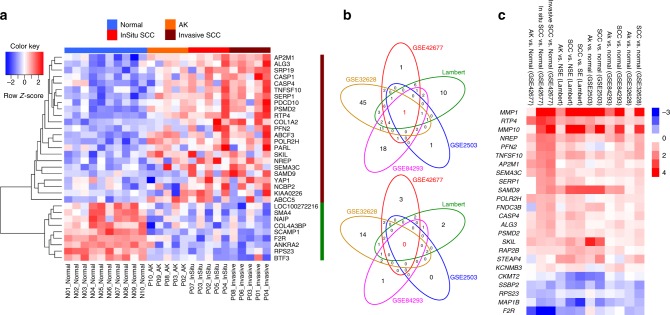
Expression profiles of genes significantly amplified or deleted in cSCC. **a** Expression heatmap of significantly amplified and upregulated genes (*n* = 23), and deleted and downregulated genes (*n* = 9) in the in situ vs. normal comparison that were also expressed across normal, AK, in situ and invasive SCC samples from GSE42677. **b** Venn diagrams of overlap across five gene expression data sets of significantly amplified genes and overexpressed (upper panel) and significantly deleted and downregulated genes (lower panel). **c** Log_2_ fold changes (FC) of pairwise comparisons of AK vs. normal and SCC vs. normal across the five data sets for 19 amplified/upregulated and 5 deleted/downregulated expressed genes shared across at least three data sets. Within the heatmap, red colour indicates the upregulation in AK or SCC compared to normal control and blue colour indicates the downregulation in AK or SCC in relation to normal skin

Within the amplified regions identified by GISTIC analysis, the mRNA levels of 23 genes were upregulated in cSCC compared to normal skin in GSE42677 (Fig. 6a), 38 in Data Set 1, 78 in GSE32628, 7 in GSE2503 and 43 in GSE84293 (Fig. 6b, top Venn diagram). Overlap analysis revealed that 19 genes were encoded in amplified regions and upregulated at the mRNA level in at least three of these data sets (Fig. 6b, c) indicating that these genes may play tumour promoting roles in cSCC. Nine genes within deleted regions identified by GISTIC analysis were also downregulated at the mRNA level in cSCC compared to normal skin in GSE42677 (Fig. 6a), 11 in Data set 1, 24 in GSE3268,, 2 in GSE2503 and 5 in GSE84293 (Fig. 6b, bottom Venn diagram). Overlap analysis revealed that five genes were encoded in deleted regions and downregulated at the mRNA level in at least three of these data sets (Fig. 6b, c) indicating that these genes may play tumour suppressor roles in cSCC.

### Identification of WD and MD/PD group-specific SMGs

**Fig. 7:**
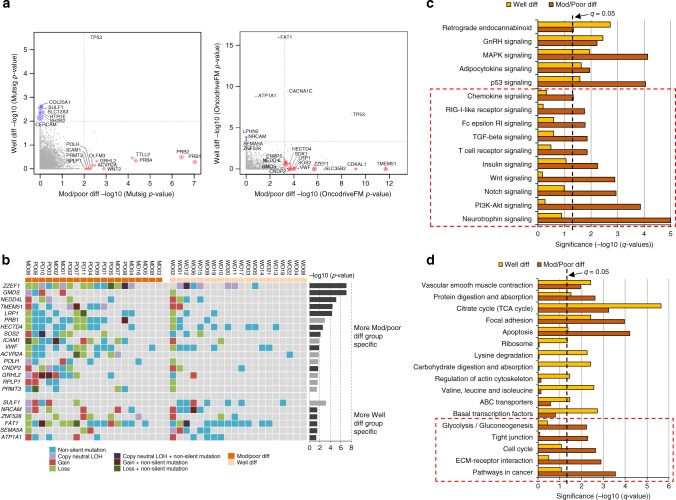
Comparison of significantly mutated genes and pathways between moderate/poor and well-differentiated groups. **a** Group-specific SMGs from the randomisation test based on MutSigCV and OncodriveFM *p*-values, respectively. Moderate/poor group-specific genes were marked with red circles, while well-differentiated group-specific genes were marked with blue circles. The size of the circle corresponds to its significance compared to that expected by chance. **b** Mutation OncoPrint of group-specific genes that were expressed across 40 cSCC samples in moderate/poor and well-differentiated groups. Significance level (-log_10_(*p*-value)) from the randomisation test was also shown for each gene as bar charts. Genes derived from the OncodriveFM statistics were shown in dark grey, while genes derived from MutSigCV statistics were in light grey. Genes at the top panel appeared to be more moderate/poor group specific, while genes at the bottom panel were more well-differentiated group specific. **c** Significantly mutated KEGG signalling pathways and **d** biological processes between moderate/poor and well-differentiated groups. Significant pathways and processes in moderate/poor group only were marked with a dotted square. Pathways and biological processes in the bar charts were sorted with terms significant in both groups at the top, and terms significant in moderate/poor group only at the bottom (indicated with red dashed boxes)

Differentiation status is a prognostic marker in cSCC (reviewed in ref. ^4^). We developed a randomisation test strategy to identify WD and MD/PD group-specific SMGs based on the MutSigCV and OncodriveFM significance *p*-values (see Methods, Fig. 7a), and further selected those group-specific genes that were also regarded as expressed in the GEP data sets. This led to 16 MD/PD-specific and 6 WD-specific SMGs, respectively (Fig. 7b). The MD/PD-specific genes included *TMEM51* and *GRHL2* previously identified in the analysis of the whole 40 exomes but also identified additional MD/PD-specific genes *ZZEF1*, *GMDS*, *NEDD4L*, *LRP1*, *PRB1*, *HECTD4*, *SOS2*, *ICAM1*, *VWF*, *ACVR2A*, *POLH*, *CNDP2*, *RPLP1* and *PRMT3*. Their significance has yet to be described in cSCC although non-silent mutations in some of these genes have been observed in cSCC^10^ (Supplementary Data 23). Interestingly, the expression of *ACVR2A* appeared to decline gradually with the disease progression, with the highest level of expression in the normal skin and the lowest in SCCs in all five GEP data sets (Supplementary Fig. 11), suggesting its potential tumour suppressing role in disease progression. The WD-specific genes included the previously identified *ATP1A1* and additionally *SULF1*, *ZNF528*, *NRCAM*, *FAT1* and *SEMA5A*. *FAT1* has been identified as a significantly mutated putative driver gene in cSCC^6,10^ and in normal sun exposed skin^6^. None of these other WD-specific genes have been associated with cSCC although non-silent mutations in some of these genes have been observed in cSCC^10^ (Supplementary Data 23).

### Mutational pathway analysis

Mutational pathway analysis revealed several KEGG signalling pathways that were more mutated in the MD/PD group, such as NOTCH, TGF-β, WNT, PI3K-AKT and neurotrophin signalling (Fig. 7c and Supplementary Data 24, 25). The MD/PD-specific mutated pathways also included those related to immune responses such as T-cell receptor, Fc epsilon RI, RIG-I-like receptor and chemokine signalling pathways (Fig. 7c). Furthermore, our mutational pathway analysis revealed that biological processes of ECM-receptor interaction, cell cycle, tight junction and glycolysis/gluconeogenesis were also more significantly mutated in the MD/PD group compared to WD group, whereas lysine degradation, regulation of the actin cytoskeleton, carbohydrate digestion and adsorption and basal transcription factors were among those specific to the WD group (Fig. 7d and Supplementary Data 25). Computational pathway analysis of mutated genes in cSCC has not previously been performed. Manual pathway assessment previously suggested involvement of the RAS/RTK/PI3K and cell cycle pathways in aggressive cSCC^10^ consistent with our observations (Fig. 7c, d). The findings that PI3K signalling is frequently altered in more aggressive SCCs such as HNSCC, lung and oesophageal SCC^11^ further supports our MD/PD pathway analysis implicating PI3K-AKT signalling in disease progression.

### Patient-derived cell lines reflect primary tumours

To facilitate future functional studies, tumour keratinocytes were cultured from multiple tumours and a panel of 15 cell lines was selected for full characterisation (Supplementary Data 26). Nine were derived from five OTR patients on immunosuppressive drugs including five from a single patient (PM1, MET1,2,4, T9)^47^. Six lines were derived from five immunocompetent patients (IC1, IC1MET, IC8, IC12, IC18 and IC19) with IC1/IC1MET being paired primary and metastatic lymph node derived lines, respectively.

**Fig. 8:**
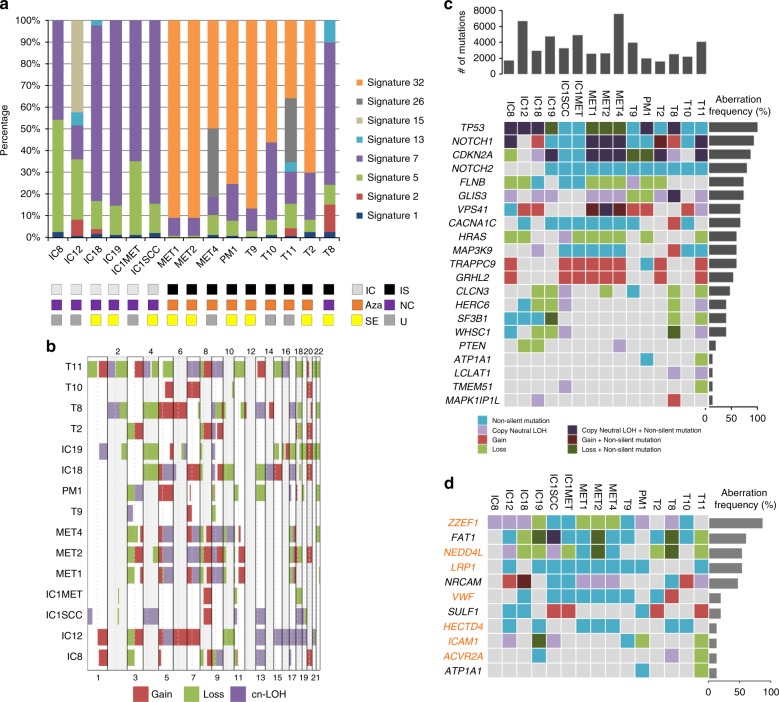
Mutation signatures, somatic CNA/LOH and mutation profiles for identified SMGs in cSCC tumour samples in SCC cell lines. **a** Mutation signatures across all SCC cell lines. Again, signature 7 and 32 were the most dominant signatures. IC immunocompetent, IS immunosuppressed, Aza confirmed azathioprine exposure, NC no confirmed azathioprine exposure, SE sun exposed site, U unknown if sun exposed site **b** OncoPrint of copy gain, loss and CN-LOH segments. **c** Mutation OncoPrint for 22 SMGs detected in cSCC tumour samples across cell lines, ordered by aberration frequency. **d** Mutation OncoPrint for WD/MD/PD driver genes detected in cSCC tumour samples across cell lines, ordered by frequency. MD/PD-specific genes found in cSCC samples are highlighted in orange

In general, mutational profiles of patient-derived cell lines were comparable with those of the tumour sample set (Supplementary Data 27). Mutation signature analysis showed that signature 7 was present in all cell lines, and eight lines from four patients also contained signature 32, all of which were derived from tumours from immunosuppressed patients receiving azathioprine (Fig. 8a and Supplementary Data 28). Interestingly, PM1, MET1, 2, 4, and T9 from the same patient show very similar mutation signature compositions, except that MET4 also exhibited signature 26 associated with defective DNA mismatch repair^14^ in 32% of its total mutations (Fig. 8a and Supplementary Data 28). OncoPrint analysis indicated that many of the cell lines have alterations in mismatch repair genes (Supplementary Fig. 12) but this does not correlate with the presence of signature 26. Analysis of somatic CNA/LOH revealed many recurrently altered regions that were also present in patient tumours including amplification at 3q26, 7q21 and 11q22, and deletions at 5q13 and 3p12 (Fig. 8b). The cell lines exhibited a high degree of chromosome instability similar to the most genomically altered primary tumours. Mutations in the 22 SMGs from the primary tumour set also frequently occurred in cell lines, with *TP53*, *NOTCH1/2* and *CDKN2A* the most recurrently altered with similar mutational profiles in primary tumour samples and cell lines (Fig. 8c, Supplementary Data 29 and Supplementary Fig. 13).

We also performed gene expression analysis of our cell lines and three separate isolates of primary normal human keratinocytes (NHKs) using Illumina Beadchip arrays (Supplementary Data 30). *NOTCH2*, a commonly downregulated SMG in the primary tumour data set, was also downregulated in cSCC cell lines in comparison to NHKs (Supplementary Fig. 14A). 2/7 commonly upregulated SMGs (*CDKN2A and WHSC1*) were also upregulated in cSCC cell lines compared to NHKs (Supplementary Fig. 14A). 3/19 commonly amplified and upregulated genes *POLR2H*, *ALG3* and *AP2M1*, were also upregulated in the cell lines relative to NHKs and 2/5 of the commonly deleted and downregulated genes, *SSBP2* and *F2R*, were also downregulated in cSCC relative to NHKs (Supplementary Fig. 14B). We further identified the mutational profiles for MD/PD and WD group-specific SMGs in cell lines, with MD/PD-specific SMGs *ZZEF1*, *NEDD4L*, *LRP1*, *VWF*, *HECTD4*, *ICAM1* and *ACVR2A* being the most frequently altered, and WD-specific genes *FAT1*, *NRCAM*, *SULF1* and *ATP1A1* recurrently altered in SCC lines (Fig. 8d). Our cell lines thus represent a well characterised resource, which reflects the molecular landscape of primary cSCC.

## Discussion

Here, we present the largest analysis of cSCC WES so far performed and provide a unique data set with 20 samples from moderately/poorly differentiated tumours, 20 from well-differentiated tumours and 15 samples from cSCC cell lines. Thirty-three primary tumour samples and nine cell lines were derived from immunosuppressed patients. Patients who are iatrogenically immunosuppressed to prevent organ transplant rejection have been recognised as being at greatly increased risk of cSCC (100**–**250-fold) with a reversal of the normal BCC:cSCC ratio from 4:1 to 1:2^2,48^.

Each biological process causing mutations in somatic cells that may drive tumour formation leaves a mutational signature and deciphering these signatures is a unique opportunity to reveal the mutational processes operative in different cancer types^13,14,16,17,49^. We applied this approach to our data set and found five previously identified signatures in our tumours including signature 7, which has been associated with exposure to UVR a known potent skin cancer carcinogen. The UVR signature was found in 33/37 exomes examined and in all 15 cell lines. A novel signature, termed signature 32 was found in 27 of the tumour samples and 8 of the cell lines (Figs. 1, 8). Interrogation of the clinical history for each patient found that the presence of signature 32 correlated with immunosuppressive regimens including use of azathioprine. Although immunosuppressants such as mycophenolate mofetil, cyclophosphamide and cyclosporine A affect DNA damage mechanisms, including UV induced DNA excision repair^50–52^, three patients received azathioprine alone and analysis of treatment times revealed a strong positive correlation with exposure time to azathioprine and signature 32 prevalence (Supplementary Fig. 3). All of the signature 32 positive cell lines were also from immunosuppressed patients who received azathioprine (Fig. 8). Furthermore, only exposure to azathioprine and not the other immunosuppressive drugs correlated with the presence of signature 32 and the only significant correlation of immunosuppressive drug use and the presence of any of the mutational signatures was with azathioprine exposure and signature 32 (Supplementary Fig. 2). This signature is likely to be of biological relevance as mutational signature analysis of mutations in the putative driver genes identified in our study, revealed that over 65% of these were signature 32 (Fig. 3c). Azathioprine, an inhibitor of de novo purine synthesis, is associated with selective ultraviolet A (UVA) photosensitivity and mutagenic effects in the skin^53^. The azathioprine metabolite 6-thioguanine (6-TG) replaces a small proportion of DNA guanine and becomes a strong UVA chromophore, interacting with UVA to generate reactive oxygen species, which cause widespread DNA damage and protein oxidation; the latter damages the DNA repair proteome increasing UVB mutagenicity^53,54^. Azathioprine photosensitivity is clinically measurable, may be associated with a specific genetic signature in basal cell carcinoma^55^ and can be reduced by switching from azathioprine to mycophenolate mofetil (MMF), although azathioprine metabolites may persist in the skin for several years after withdrawal^56^. Given the photosensitising properties of azathioprine we postulate that the combined action of UVR and incorporation of azathioprine metabolites into DNA provide the biochemical basis for this novel signature. We propose that these azathioprine effects are associated with this new genetic signature (signature 32) that contributes to tumour progression. This has clinical significance for the many patients currently receiving azathioprine who should be counselled about their skin cancer risk and UVR photosensitivity.

We confirm the high mutational burden present in cSCC with an average of over 1700 mutations present in the primary tumour exomes (Fig. 1). WES data enabled us to assess clonality and revealed that most tumours are highly heterogenous containing 2**–**11 clones (Fig. 4). GISTIC analysis revealed common areas of copy number loss and gain in our samples with MD/PD tumours exhibiting higher levels of chromosomal instability compared to WD tumours (Fig. 2) further illustrating the genetic complexity of cSCC.

We employed three computational methods (MutSig, OncodriveClust, OncodriveFM) and identified 351 SMGs at the cutoffs we used. Twenty-two genes were found to be significant in two or more of these programmes enabling a further ranking of the potential driver genes (Fig. 3). Combination of these analyses with CNA/LOH analysis indicate that alteration of *NOTCH1* and *NOTCH2* genes are among the most common events in cSCC (Fig. 3) with only 3/20 MD/PD tumours and 5/20 WD tumours escaping alteration in either of these genes indicating that the NOTCH pathway is an almost universal tumour suppressor pathway in cSCC probably due to its role in promoting cell cycle exit and differentiation^57^. We also identify *TP53* alterations in 70% of our tumours and *CDKN2A* changes in nearly 50% of cases. Clonality analysis revealed that *CDKN2A* mutations are clonal (Fig. 4c, d) indicating a likely early event in formation of the tumours in which they were identified. Strikingly, despite deep sequencing efforts, *CDKN2A* mutations in contrast to *TP53* or *FAT1* mutations have yet to be identified in sun-exposed ‘normal skin’^6^ suggesting a key gatekeeper role of p16INK4B in cSCC. Our findings also suggest that mutations in *ATP1A1*, *HERC6*, *MAPK1P1L*, *GRHL2*, *TRAPPC9*, *FLNB*, *NOTCH1* and *MAP3K9* represent early driving events in cSCC (Fig. 4c, d). Within these, alteration in *ATP1A1* may pre-dispose to well-differentiated tumours, whereas alterations in *GRHL2* may pre-dispose to more poorly differentiated and potentially poorer prognosis tumours (Fig. 7). *GRHL2* mutations are associated with an autosomal-recessive ectodermal dysplasia^58^ and this transcription factor may drive a cancer stemness phenotype in oral SCC^59^. This gene clearly warrants further investigation in cSCC.

To further sift our list of somatically mutated genes and those that are encoded within regions of common amplification or loss we integrated our WES data with five independent gene expression data sets (Figs. 5, 6). Interestingly, despite the proposed tumour suppressor role of inactivating mutations in *CDKN2A* we consistently observed upregulation of *CDKN2A* gene expression in the GEP data sets and in our cell lines (Fig. 5 and Supplementary Fig. 14). Transcription factor motif analysis indicates that ERK signalling through ETS transcription factors may be operative during SCC progression^7^ and this may act to upregulate CDKN2A expression as part of a stress induced senescence programme^60^ possibly explaining this discrepancy. This analysis highlights potential oncogenic tumour promoting roles for the actin binding protein encoded by *FLNB* and the histone methyltransferase encoded by *WHSC1*, as expression of both of these genes are elevated during cSCC progression and they do not contain premature termination codon or splice site mutations (Fig. 5 and Supplementary Fig. 6, 7). We also identified genetic alteration of the arginine methyltransferase, *PRMT3*, specifically in the MD/PD tumours (Fig. 7B), further indicating that epigenetic modifiers may regulate cSCC progression. Genes that are amplified and upregulated during cSCC progression may act as oncogenic drivers of disease and we revealed 19 further genes with this potential role including the SKI like proto-oncogene SnoN encoded by the *SKIL* gene (Fig. 6). This transcription factor can modulate canonical TGF-β signalling^61^ and pathway analysis revealed that genomic alterations in TGF-β signalling are enriched in the MD/PD group of tumours (Fig. 7C). Consistent with this we also identify alterations in the *ACVR2A* gene that encodes for an activin/BMP receptor enriched in this tumour group, which is also downregulated during tumour progression (Fig. 7B and Supplementary Fig. 11) implying a tumour suppressor role for this gene. Our recent studies have confirmed that the TGF-β pathway acts as a potent tumour suppressor pathway in cSCC with mutational inactivation of *TGFBR1* and/or *TGFBR2* occurring in ~40% of cSCC tumours^15^.

Integration of gene expression data sets with GISTIC analysis of CNVs provides a powerful technique for identifying genes, which contribute to disease progression without the involvement of SNV alterations. This analysis implicates *SEMA3C*, *STEAP4*, *MMP10*, *RAP2B* and *AP2M1* as a potential cSCC drivers (Fig. 6). These genes have been implicated in promoting tumour growth. SEMA3C can activate RTK signalling and drive prostate cancer growth^62^, STEAP4 may promote colorectal cancer development^63,^ MMP10 may mediate c-Fos driven cSCC development^64^, RAP2B is a well described oncogenic activator^65^ and AP2M1 may participate in senescence escape^66^. These observations support the hypothesis that our integrated analysis approach has potentially revealed novel drivers of cSCC and provides further impetus for functional interrogation of the genes and pathways revealed in our study.

In summary, this study describes the complex molecular landscape of cSCC and identifies new potential driver genes, pathways and processes associated with both the development of well-differentiated and potentially poorer prognosis moderately/poorly differentiated tumours in both immunosuppressed and immunocompetent patients. Importantly we identify a novel mutational signature associated with chronic azathioprine exposure and describe the molecular landscape of 15 cSCC cell lines derived from both primary and metastatic lesions which reflect the complexity of tumours and provides a unique resource for determining the biological significance of the molecular events responsible for cSCC maintenance and progression.

## Methods

### Collection of patient samples

The study was approved by the East of Scotland Research Ethics Service (reference: 08/S1401/69) and the East London and City Health Authority Local Ethics Committee and conducted according to the Declaration of Helsinki Principles. All patients participating in the study provided written, informed consent. Punch biopsies of cSCC were collected in tissue culture transport medium comprising DMEM supplemented with 50 units/ml penicillin and 50 μg/ml streptomycin and 1x fungizone solution (all from Life Technologies, Paisley, UK) or snap-frozen in liquid nitrogen. Venous blood samples were taken from patients into EDTA tubes, aliquoted into cryovials and stored at −80 °C. Histology of patient samples were reviewed blind by two independent pathologists. Details of prescribed medications including drug, dose and duration were derived from two independent reviews of available clinical notes and transplant databases for all immunosuppressed patients. The estimated time of azathioprine therapy for all patients with a history of azathioprine use were calculated and rounded to the nearest month. Complete clinical notes spanning the entire patient journeys were not available for all patients and those without documentary evidence of azathioprine exposure were classified as no confirmed exposure.

### Isolation and culture of cSCC lines

Cultures were established according to a previously described method^67^. In brief, tumour tissue was cut into small pieces and dissociated by incubation at 37 °C in 0.25% trypsin-EDTA (Life Technologies, Paisley, UK) followed by physical disruption using needles. Residual tissue was removed by passing the cell suspension through a 100 μm cell strainer and SCC keratinocytes recovered by centrifugation and plated onto a mouse 3T3 feeder cell layer in keratinocyte culture medium comprising DMEM/Ham’s F12 (3:1; Life Technologies, Paisley, UK) supplemented with 10% fetal bovine serum (FBS; Biosera, Ringmer, UK) and a cocktail of mitogens^67^. Cultures were maintained in keratinocyte culture medium supplemented with 10 ng/ml EGF (Serotec, Oxford, UK) and passaged upon attaining 80% confluence. All cell lines were routinely screened for mycoplasma and were negative throughout the study.

### DNA extraction and genetic analysis

DNA was extracted from snap-frozen tissue, cultured cells and blood using the Qiagen DNA Mini kit (Qiagen, Crawley, UK) according to the manufacturer’s instructions. Quantitation was performed using the double-stranded DNA-specific Qubit dsDNA BR assay kit in conjunction with the Qubit 2.0 fluorometer (both Life Technologies, Paisley, UK). Short tandem repeat (STR) genotyping was performed to authenticate the unique identity of each cell line. Sixteen loci distributed across the human genome were amplified using the AmpFLSTR Identifiler PCR Amplification Kit followed by capillary sequencing on the 3730xl DNA analyser (all reagents from Life Technologies, Paisley, UK). Microarray-based single-nucleotide polymorphism (SNP) analysis was performed by subjecting 500 ng DNA from each cell line and matched blood to the Genome-Wide Human SNP Nsp/Sty 6.0 assay (Affymetrix, Santa Clara, CA) according to the manufacturer’s instructions. Whole-exome sequencing (WES) was performed by Oxford Gene Technology (OGT) using Agilent SureSelect All Exon v5 for exome capture. Briefly, 1 μg of DNA from each sample were used to prepare the sequencing library through shearing of the DNA followed by ligation of sequencing adaptors. Sequencing was performed on the Illumina HiSeq platform. Paired-end sequencing (2 × 100 bp) was carried out using HiSeq sequencing instruments.

### WES data processing, somatic variant calling and annotation

Thirty previously published sporadic cSCC samples^5,15^ were included and reanalysed with our ten newprimary tumour samples and 15 cSCC cell lines. WES data of all samples (except the T2 cell line, see below) were analysed using our established pipeline^68,69^. The minimum coverage for identified variant sites was required to be as ten reads. For the cell line sample T2, the variant calling was performed using the VarScan2 somatic calling module^70^. There was around 22% of DNA in the corresponding normal sample that was tumour DNA, suggested by the variant allele frequency (VAF) density from the normal sample. Within VarScan2 somatic calling, allele frequencies from the T2 cancer cell line and the normal control sample were compared by Fisher’s Exact Test, and somatic *p*-values were derived. High-confidence somatic calls were subsequently extracted for T2 using the VarScan2 ‘process-Somatic’ module, with mutations with > 20% VAFs further selected. Identified somatic variants, including single-nucleotide variants (SNV) and indels, in 40 patient samples and 12 cell lines were further annotated using Oncotator^71^ and SNPnexus^72^. Mutation signatures across the 40 exomes were identified based on the non-negative matrix factorisation (NMF) approach previously described^13^. WES data of 40 cSCC samples have been deposited to the European Genome-phenome Archive (EGA) under the accession of EGAS00001002612.

### Identification of mutational driver genes and pathways

We used three methods to identify different positive selection signals that occurred in driver mutations: OncodriveFM^27^, OncodriveCLUST^28^, both implemented in the IntoGen software^73^, and MutSigCV^26^. For OncodriveFM and OncodriveCLUST, we used corrected *p*-values (*q* < 0.05) for significance. For MutSigCV, due to the small sample size, *p*-values could not be adjusted, so raw *p* < 0.05 was applied. Overlapped genes that were identified being significant by at least two of the three methods were further selected. Significantly mutated pathways were also identified using OncodriveFM (*q* < 0.05).

### Randomisation test to identify MD/PD and WD-specific drivers

To identify moderate/poor and well-differentiated specific driver genes, we further applied OncodriveFM and MutSigCV on 20 moderate/poor and 20 well-differentiated cSCC samples separately. The significance *p*-values derived from the two methods were (-log_10_) transformed and compared between the two groups to highlight group-specific genes. In the MutSigCV test, we selected genes with *p* *<* 0.01 in one group but *p* > 0.1 in the other group. In the OncodriveFM test, we focused on genes with corrected *p* < 0.05 in one group but corrected *p* > 0.1 in the other. For these selected candidate genes, we further developed a randomisation test procedure where the pool of 40 SCC samples was randomly split into two groups of equal size (*n* = 20). We then applied MutSigCV and OncodriveFM on the two groups and recorded the significance values for our candidate genes. We repeated this randomisation procedure 100 times and the transformed *p*-values from MutSigCV or OncodriveFM generally followed a normal distribution for each gene. We next calculated the probability that the observed significance in moderate/poor or well-differentiated group was stronger than that expected by chance for each candidate. Significant genes from the randomisation test (two-tail *p* < 0.05) were further noted and regarded as the true moderate/poor or well-differentiated specific genes.

### Identification of CNA and LOH using WES data

Analyses for CNA and LOH events from WES data were based on a combinational approach previously described^69,74^. We adopted two independent approaches (ASCAT^75^ and VarScan2/DNAcopy R packages) to analyse matched tumour and normal samples for copy number analyses. Using ASCAT, a gain (amplification) is called if (predicted total copy number – predicted ploidy) > 0.6 and a loss if (predicted total copy number – predicted ploidy) < −0.6. Using DNAcopy circular binary segmentation (CBS) segments, the logR ratio was used to discriminate copy number gains and losses (logR ratio > 0.15 = gain, logR < −0.15 = loss). We then selected only the consensus calls across the two methods. In addition, we visually inspected all CNA/LOH events using the logR and B-allele frequency (BAF) plots, and further included copy number gain and loss events when they were clearly supported by one method, but just below the cutoff threshold in the other. Genes targeted by copy gain, loss and copy-neutral LOH (cn-LOH) in each sample were further identified.

### Identification of significantly amplified/deleted regions

To further identify significantly amplified and deleted regions in cSCC genomes, we applied GISTIC2.0^25^ (*q* < 0.1) using the CNA segments and markers generated by VarScan variant calling and ASCAT R package^75^. Thirty-one of the 40 cSCC exomes that passed the ASCAT analysis were used. Regions that overlap with centromere and telomere were excluded. We further removed regions that were identified as both significantly amplified and deleted.

### Tumour subpopulation identification and clonality analysis

EXPANDS^39^ was used to estimate clonal expansions and cellular frequency of each clonal and subclonal population. Somatic SNVs, indels and CNA segments derived from DNAcopy CBS algorithm were used as input for each tumour. Tumour samples with less than 200 somatic mutations were excluded from this analysis, since stable results may not be solved for these samples, resulting in processing of 35 out of the 40 WES samples. Within each tumour sample, the largest clone was identified as the dominant clone and clones with lower cellular frequencies were regarded as sub-clones. Tumour purity was also estimated based on the cellular frequency of the dominant clone. All somatic variants were assigned to their nested clones. We also used SciClone^40^ to estimate mutational clusters and Infer clonal architecture for 35/40 cSCC exomes. Similar to EXPANDS, Somatic SNVs, indels with their VAFs along with CNA segments were used as input. The cluster with the largest number of mutations was regarded as the dominant clone for each sample, and subpopulations and tumour purity were subsequently derived. The clonality results and tumour purity from both methods were further compared and assessed.

### Gene expression microarray data analysis and integration

Five sets of expression microarray data of patient samples were selected and downloaded from Gene Expression Omnibus (GEO), GSE42677^45^, GSE45216^44^, GSE2503^46^, GSE84293^7^ and GSE32628^21^. The GSE42677 data set included 10 normal skin, 5 AK, 5 in situ and 5 invasive SCC samples, the GSE2503 data set included 6 normal skin, 4 AK and 5 SCC samples, the GSE84293 data set included 8 normal skin, 9 AK and 9 SCC samples and the GSE32628 data set included 13 normal skin, 14 AK and 15 SCC samples. The available normalised expression values in log_2_ scale were used. The GSE45216 data set had 10 AK and 30 SCC. For this set, we further included 16 non-sun-exposed (NSE), 20 sun-exposed (SE) normal skin and 18 AK samples profiled using the same platform, Affymetrix Human Genome U133 Plus 2.0 Array. All raw.CEL files of 94 samples were assembled and processed together. Quality control (QC) and normalisation were performed on our O-miner transcriptomics analysis platform^76^, where the RMA (robust multi-array average) approach was used. For all five data sets, differential expression (DE) analyses between different groups were performed using limma R package^77^. The DE genes were identified using the adjusted *p*-value < 0.05.

For SMGs, as well as significantly amplified and deleted genes identified above, we further investigated their expression profiles across normal skin, AK and SCC samples. Based on the normalised non-log_2_ transformed expression values, we regarded probes with the expression values <100 as not being expressed or expressed at a low level. Thus, we further identified genes that were expressed across normal skin, AK and SCC samples in the five data sets. We then categorised them as genes with an activating role in the tumour development if they were significantly upregulated, or genes with a suppressing role if they were significantly downregulated in SCC/AK samples compared to normal skin, in at least three out of the five data sets.

For patient-derived cell lines, gene expression data were obtained from Illumina HumanHT-12 v4 Expression BeadChip. Raw image files were first processed using Illumina GenomeStudio, and then normalised using the O-miner platform where lumi R bioconductor package^78^ was applied. The limma R package was used for the DE analysis. The raw and normalised data have been deposited at GEO under the accession number of GSE98780.

## Electronic supplementary material

Supplementary Information

Description of Additional Supplementary Files

Supplementary Data 1

Supplementray Data 2

Supplementary Data 3

Supplementary Data 4

Supplementary Data 5

Supplementary Data 6

Supplementary Data 7

Supplementary Data 8

Supplementary Data 9

Supplementary Data 10

Supplementary Data 11

Supplementary Data 12

Supplementary Data 13

Supplementray Data 14

Supplementary Data 15

Supplementary Data 16

Supplementary Data 17

Supplementary Data 18

Supplementary Data 19

Supplementary Data 20

Supplementary Data 21

Supplementary Data 22

Supplementary Data 23

Supplementary Data 24

Supplementary Data 25

Supplementary Data 26

Supplementary Data 27

Supplementary Data 28

Supplementary Data 29

Supplementary Data 30

## Data Availability

All data is provided in Supplementary Data 1-30, in GSE98780 and EGAS00001002612.
