# Supplementary material for: The genomic landscape of cutaneous SCC reveals drivers and a novel azathioprine associated mutational signature
Source: Nat Commun. 2018 Sep 10;9:3667. doi: 10.1038/s41467-018-06027-1 (PMC6131170; doi:10.1038/s41467-018-06027-1)
Supplement: Supplementary file 1 — Supplementary Information [file 41467_2018_6027_MOESM1_ESM.pdf]

SUPPLEMENTARY INFORMATION

The genomic landscape of cutaneous SCC reveals drivers and a novel azathioprine associated mutational signature

Gareth J Inman et al.

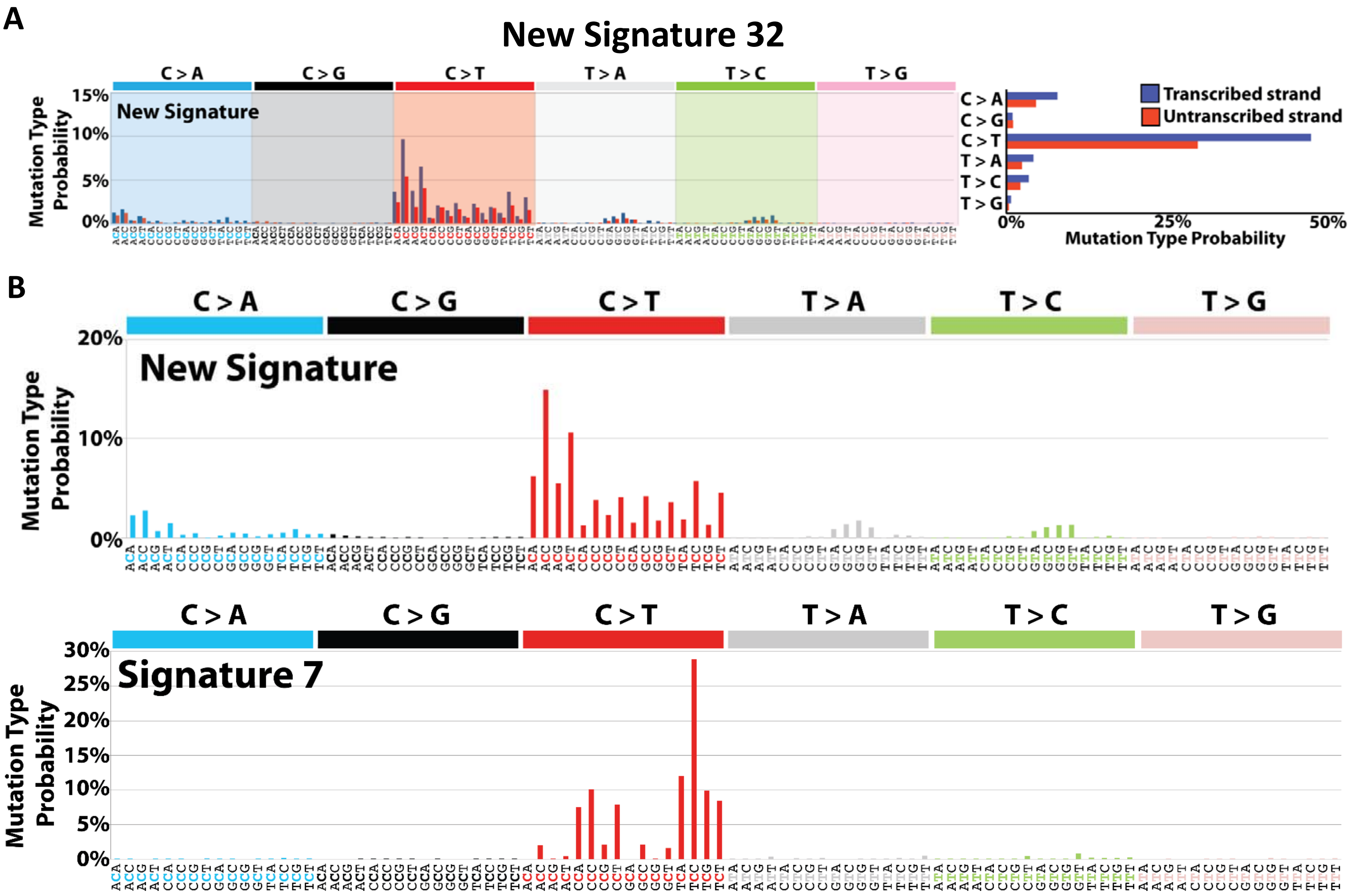

Supplementary Figure 1. Novel signature 32. It is predominately C>T mutations (75%) in combination with C>A, T>A, and T>C mutations. There seems to be a clear presence for C>X at Ap<u>C</u>pN and T>X at Gp<u>T</u>pN. This novel mutational signature exhibits a very strong transcriptional strand bias potentially indicating an interplay with TC-NER due to adducts on guanine and adenine (A). Compariosn between Signature 32 and Signature 7 which is induced by UV exposure (B).

Supplementary Figure 2

a)

Immunosuppressant History		Presence of signature 32 mutations: No	Presence of signature 32 mutations: Yes	Fisher's Exact Test P-value (Exact Sig. 2-sided)
**Azathioprine**	No	8	1	**<0.0001**
	Yes	2	26	
**Prednisolone**	No	6	7	0.1178
	Yes	4	20	
**Ciclosporin**	No	6	9	0.2578
	Yes	4	18	
**Mycophenolate Mofetil**	No	8	23	0.6527
	Yes	2	4	
**Cyclophosphamide**	No	10	26	>0.9999
	Yes	0	1	

Fisher's exact test for association between history of immunosuppressant drugs and presence of mutational signatures in individual tumours.

a) Matrix of numbers of tumours derived from patients and their exposure to immunosuppressant drugs and the presence of signature 32 with associated Fisher's exact p values.

b) Table showing the Fisher's exact p values from analysis of associations of all immunosuppressive drug exposures and all identified mutational signatures. Only a history of azathioprine treatment is associated with the presence of signature 32 P<0.001. (Bonferroni correction for multiple tests, association is considered significant when p < 0.0017.

b)

	Fisher's Exact Test P-value (Exact Sig. 2-sided)					
	Signature					
**Immunosuppressant**	**1**	**2**	**5**	**7**	**13**	**32**
**Azathioprine**	>0.9999	0.1405	>0.9999	>0.9999	0.1405	**<0.0001**
**Prednisolone**	>0.9999	0.2777	>0.9999	>0.9999	0.2777	0.1178
**Ciclosporin**	>0.9999	0.0586	>0.9999	0.2830	0.0586	0.2578
**Mycophenolate Mofetil**	>0.9999	>0.9999	>0.9999	0.5236	>0.9999	0.6527
**Cyclophosphamide**	>0.9999	>0.9999	>0.9999	>0.9999	>0.9999	>0.9999

A

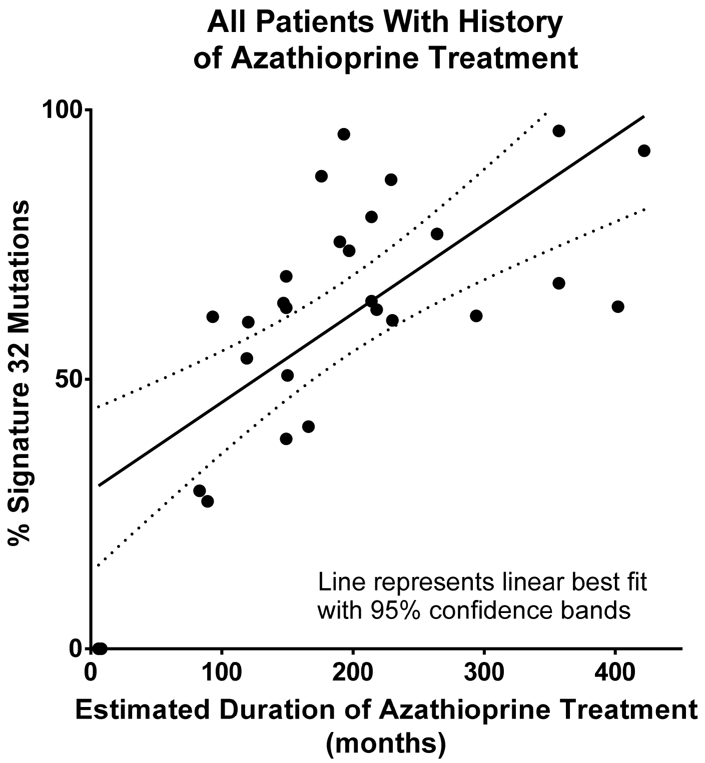

B

Sample	Estimated Duration of Azathioprine Treatment (months)	% Signature 32 Mutations
MD01	149	38.94%
MD02	150	50.72%
MD04	193	95.49%
MD05	422	92.41%
MD06	402	63.47%
MD16	197	73.87%
PD01	149	63.29%
PD03	149	69.11%
PD04	93	61.62%
PD05	147	64.15%
PD07	294	61.76%
PD08	166	41.22%
PD09	119	53.88%
PD10	6	0.00%
PD11	264	76.98%
WD01	8	0.00%
WD03	89	27.35%
WD05	230	60.95%
WD06	190	75.50%
WD09	120	60.60%
WD11	83	29.32%
WD12	214	80.15%
WD13	357	67.84%
WD14	214	64.52%
WD17	218	62.92%
WD19	357	96.11%
WD20	176	87.71%
WD21	229	87.06%

C

Correlation

			% Signature 32 Mutations
Spearman’s rho	Estimated Duration of Azathioprine Treatment (months)	Correlation Coefficient	0.679
		Sig. (2-tailed)**	p < .0001
		N	28

** Correlation is significant at the 0.01 level (2-tailed).

Supplementary Figure 3. Duration of azathioprine treatment correlates with intensity of signature 32.
A. Scatterplot of % of signature 32 mutations against estimated duration of azathioprine treatment. B. Table of samples from patients with history of azathioprine use detailing estimate of azathioprine treatment duration (months) and percentage of signature 32 mutations detected in the tumours. C. Spearman's rank-order correlation revealing a statistically significant positive correlation $r_s(26)=0.679$, $p < 0.0001$.

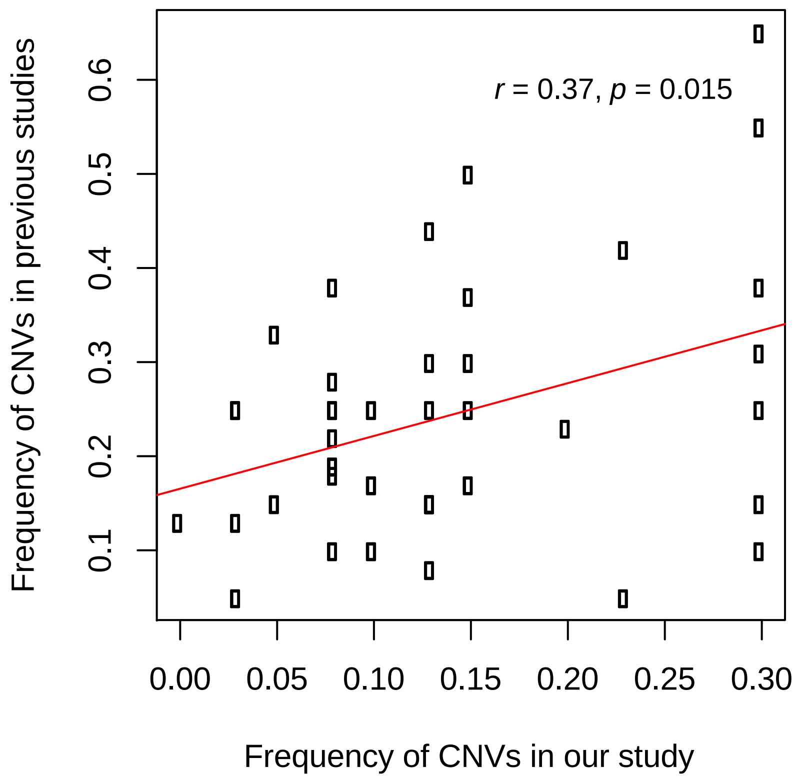

Supplementary Figure 4. Significant positive correlation between this study and previous studies for top frequent CNVs. Previous CNV studies included Salgado et al. Genes, Chromosomes and Cancer 2010, Sekulic et al. Cancer Prev Res 2010, Hameetman et al BMC Cancer 2013 and Purdie et al JID 2009

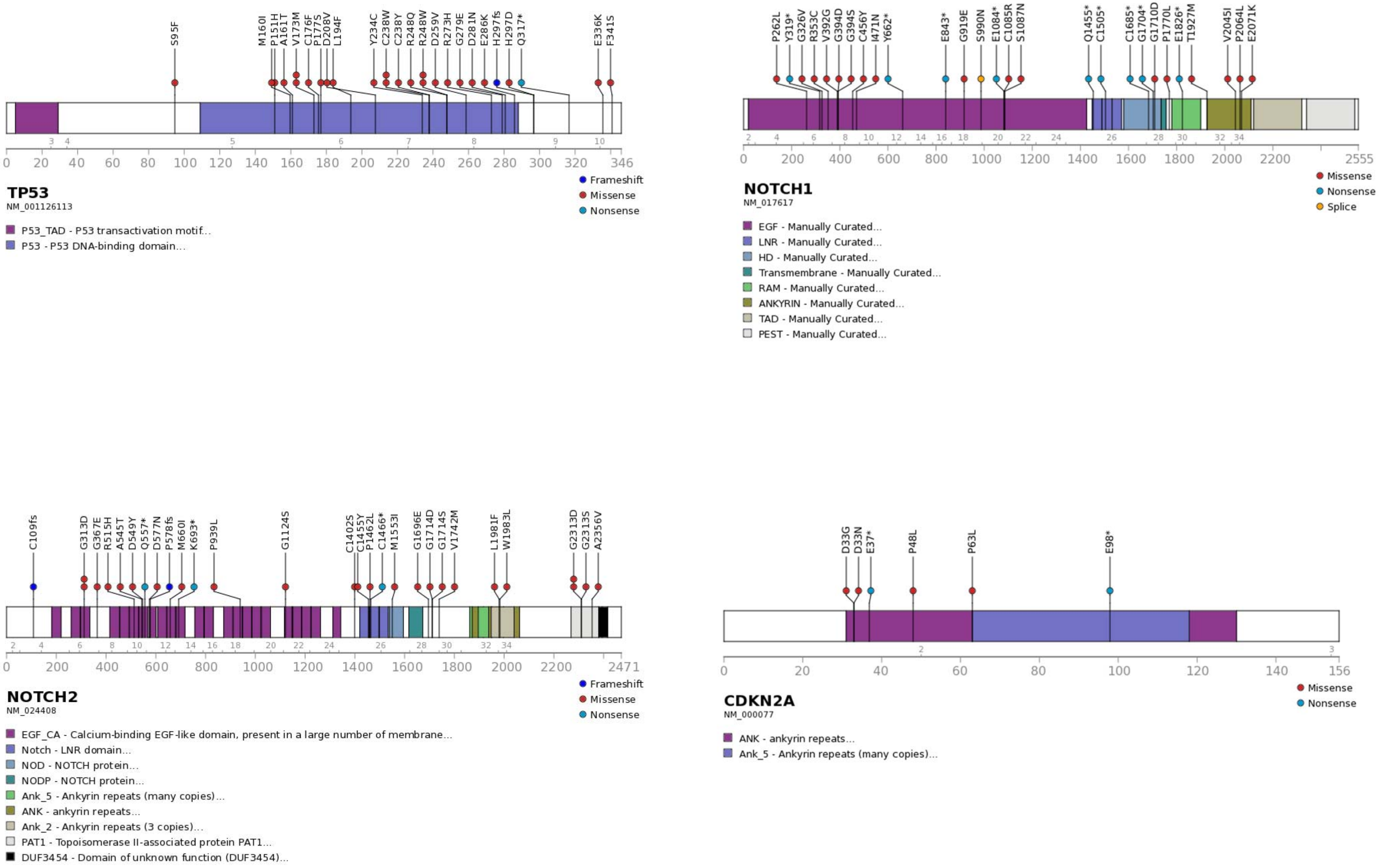

Supplementary Figure 5. Mutational spectrum in 4 SMGs, TP53, NOTCH1, NOTCH2 and CDKN2A

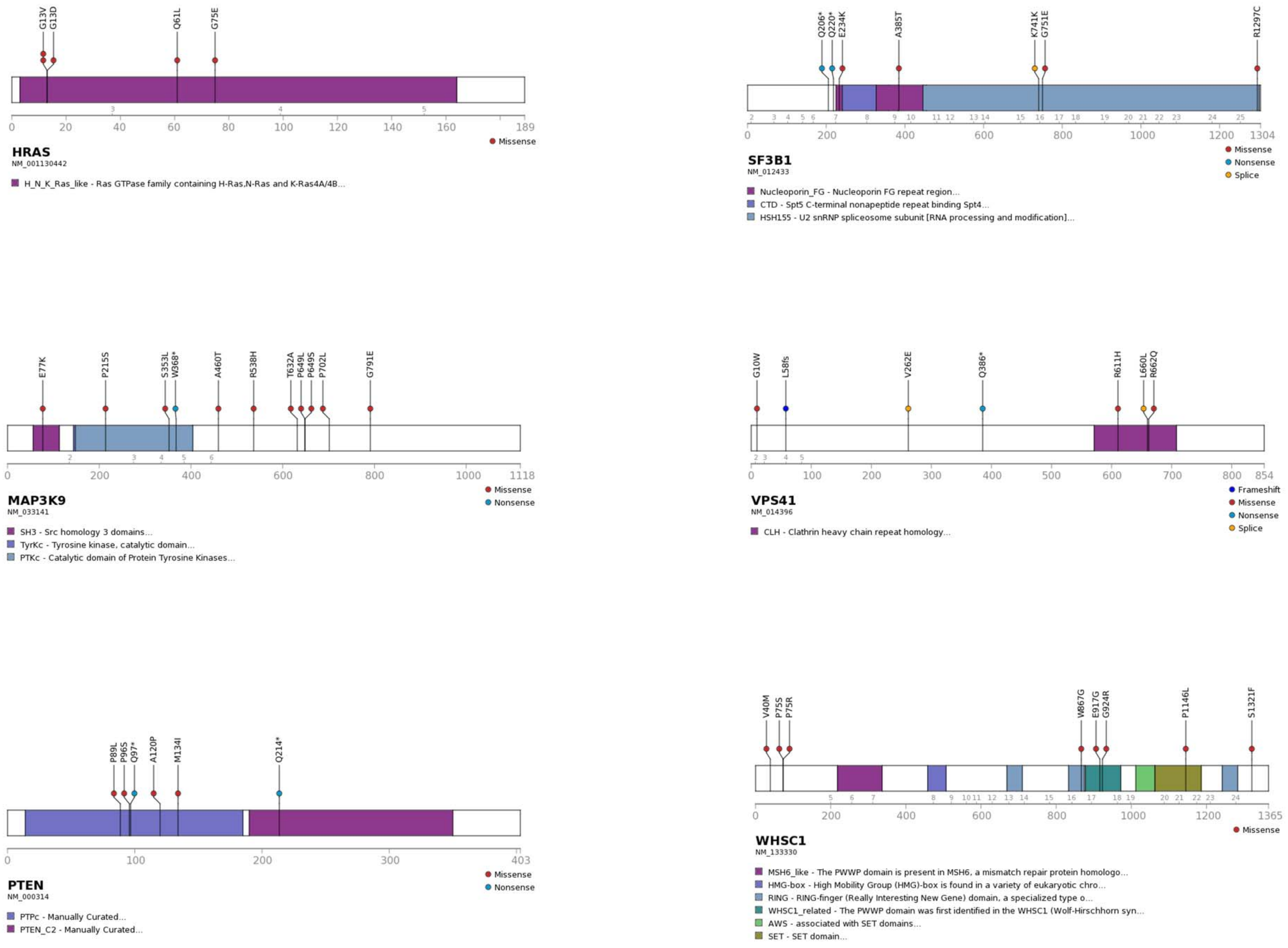

Supplementary Figure 6. Mutational spectrum in HRAS, MAP3K9, PTEN, SF3B1, VPS41 and WHSC1

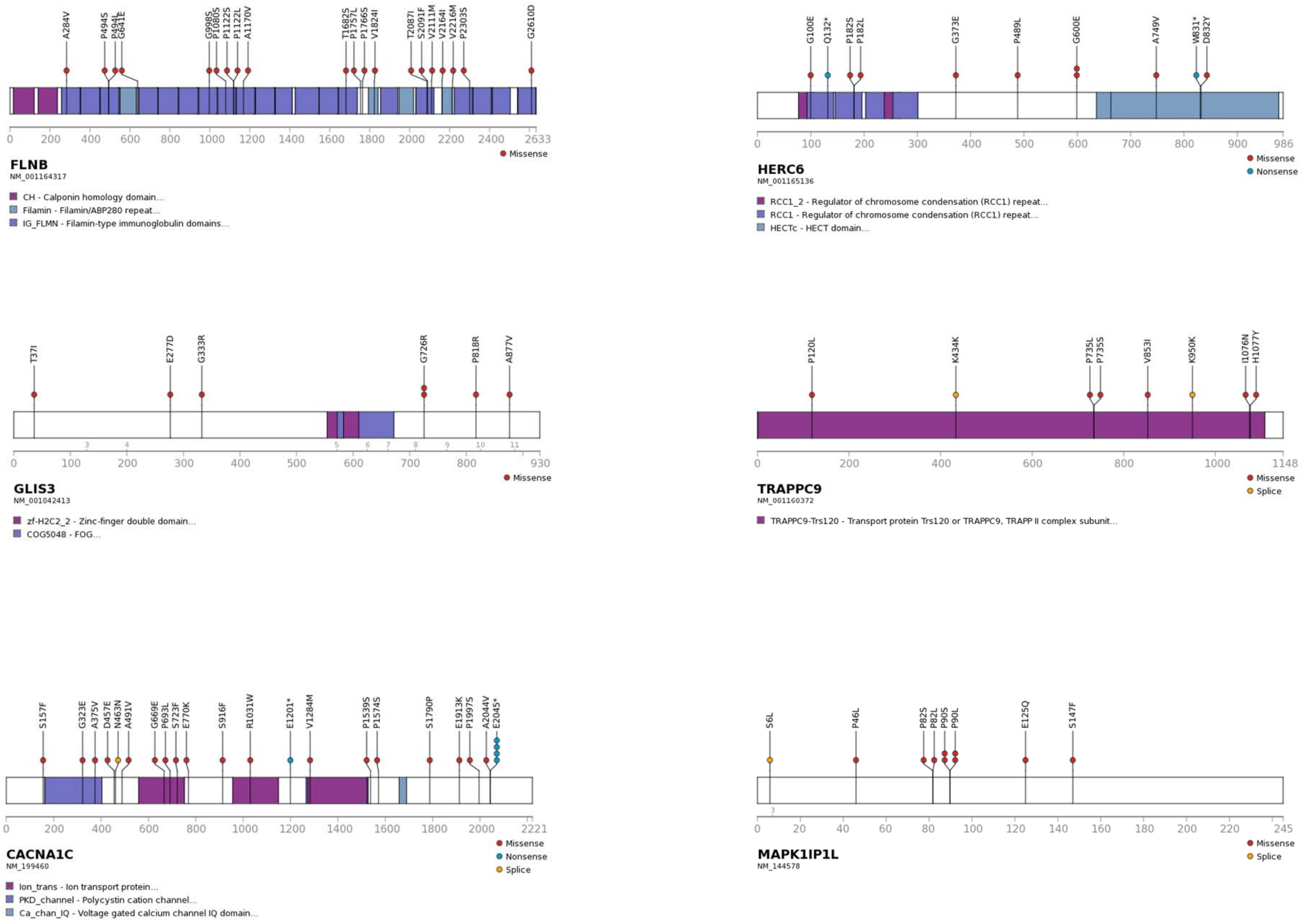

Supplementary Figure 7. Mutational spectrum in FLNB, GLIS3, CACNA1C, HERC6, TRAPPC9 and MAPK1P1L

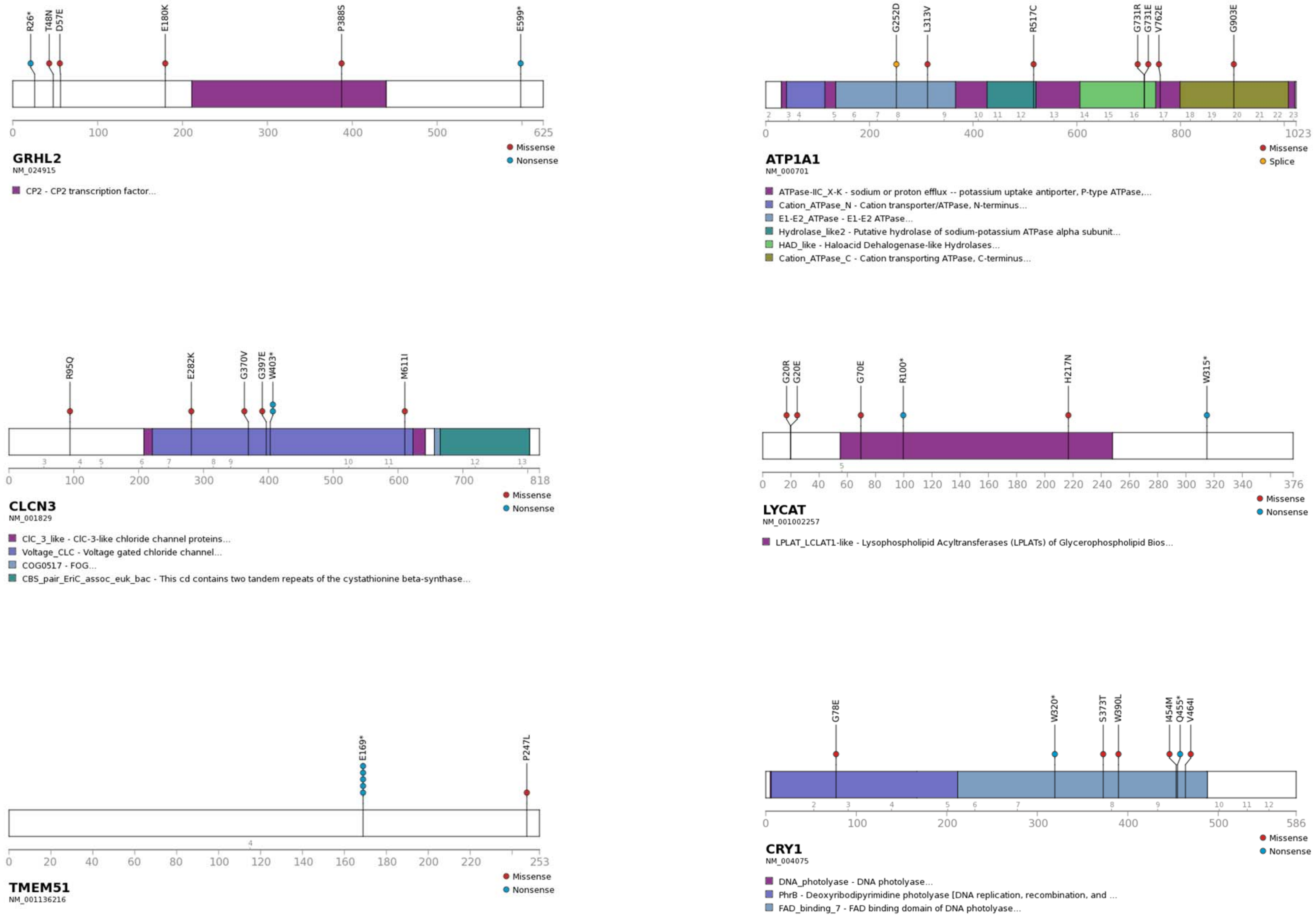

Supplementary Figure 8. Mutational spectrum in GRHL2, CLCN3, TMEM51, ATP1A1, LCLAT1 and CRY1

Supplementary Figure 9. Correlation of tumour purity estimated by EXPANDS and SciClone

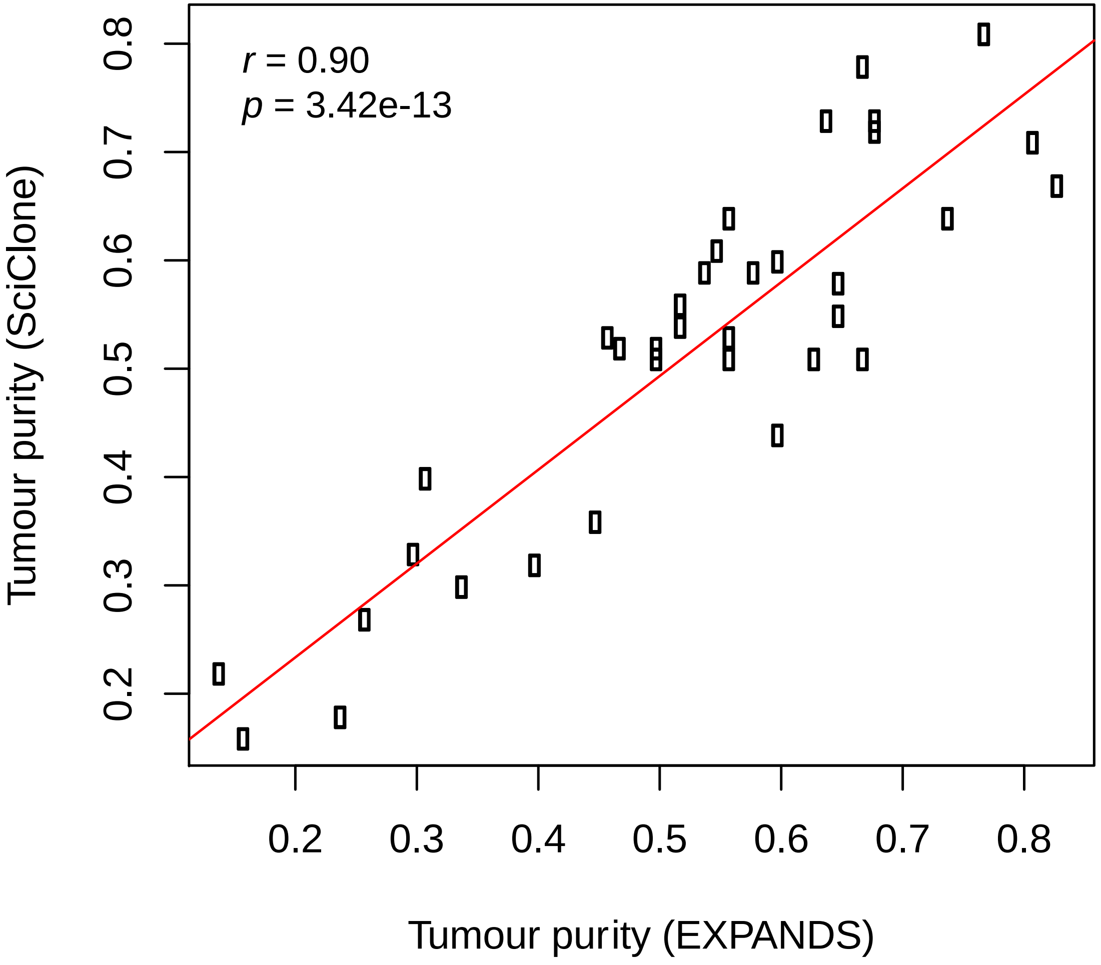

Supplementary Figure 10. Comparison between EXPANDS and SciClone across 35/40 cSCC whole exomes

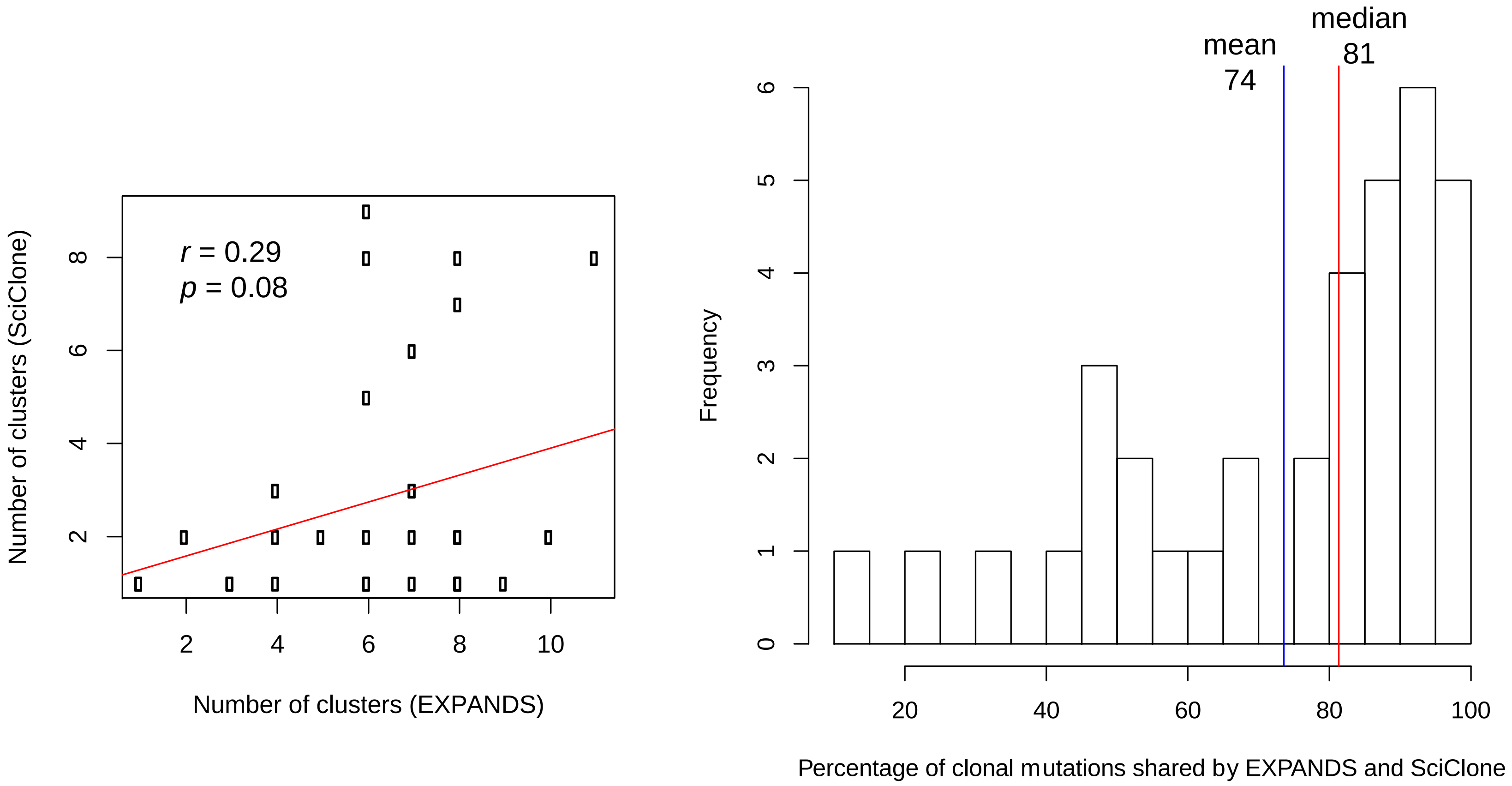

Overall, 74% all 'clonal' mutations called by EXPANDS were also called 'clonal' by SciClone

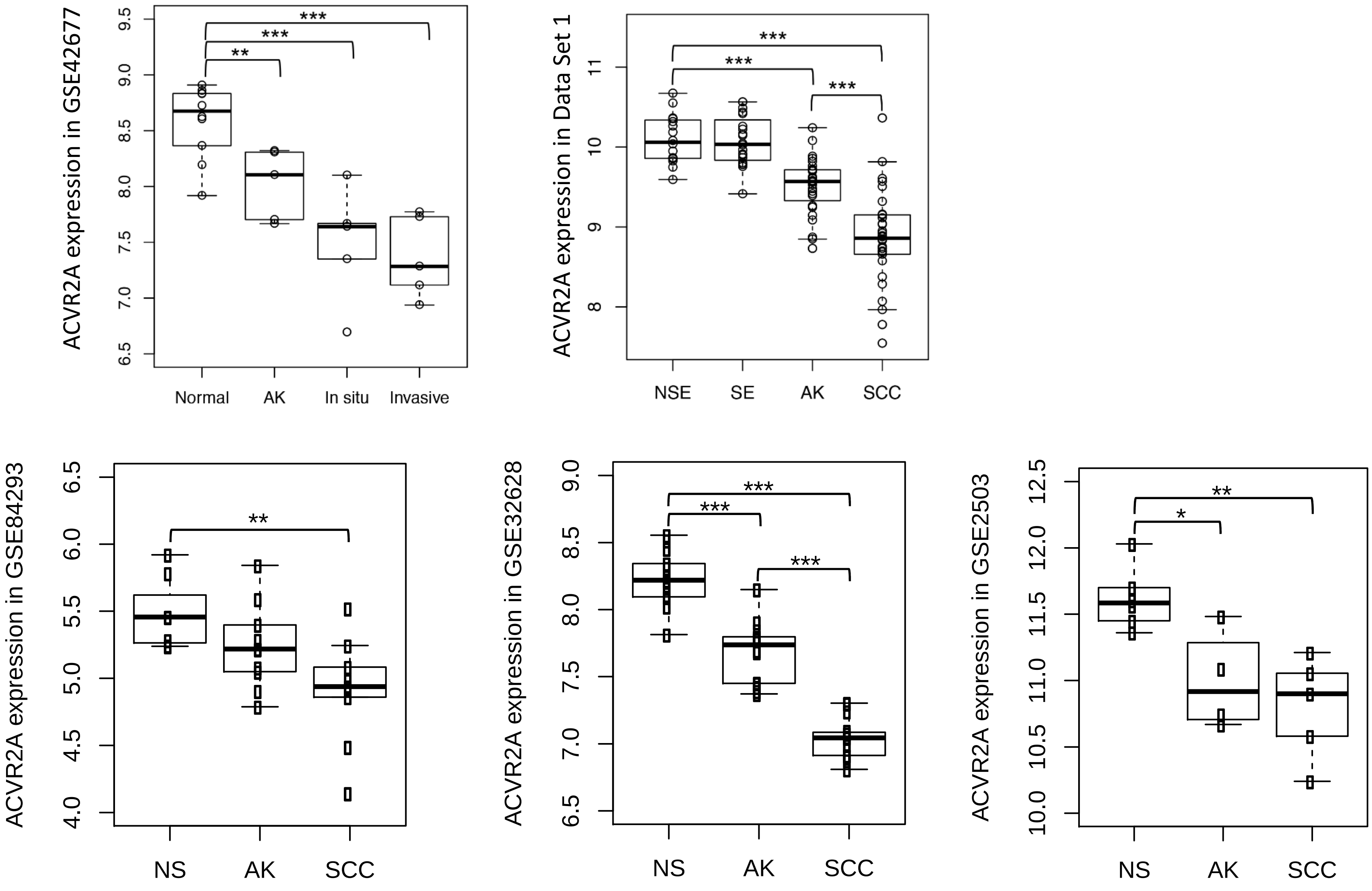

Supplementary Figure 11. ACVR2A expression across NS, AK and SCC groups in 5 gene expression profile (GEP) data sets, GSE42677, GSE45216 plus additional samples (Data set 1), GSE84293, GSE32628 and GSE2503 reveals a significant decrease with disease progression.

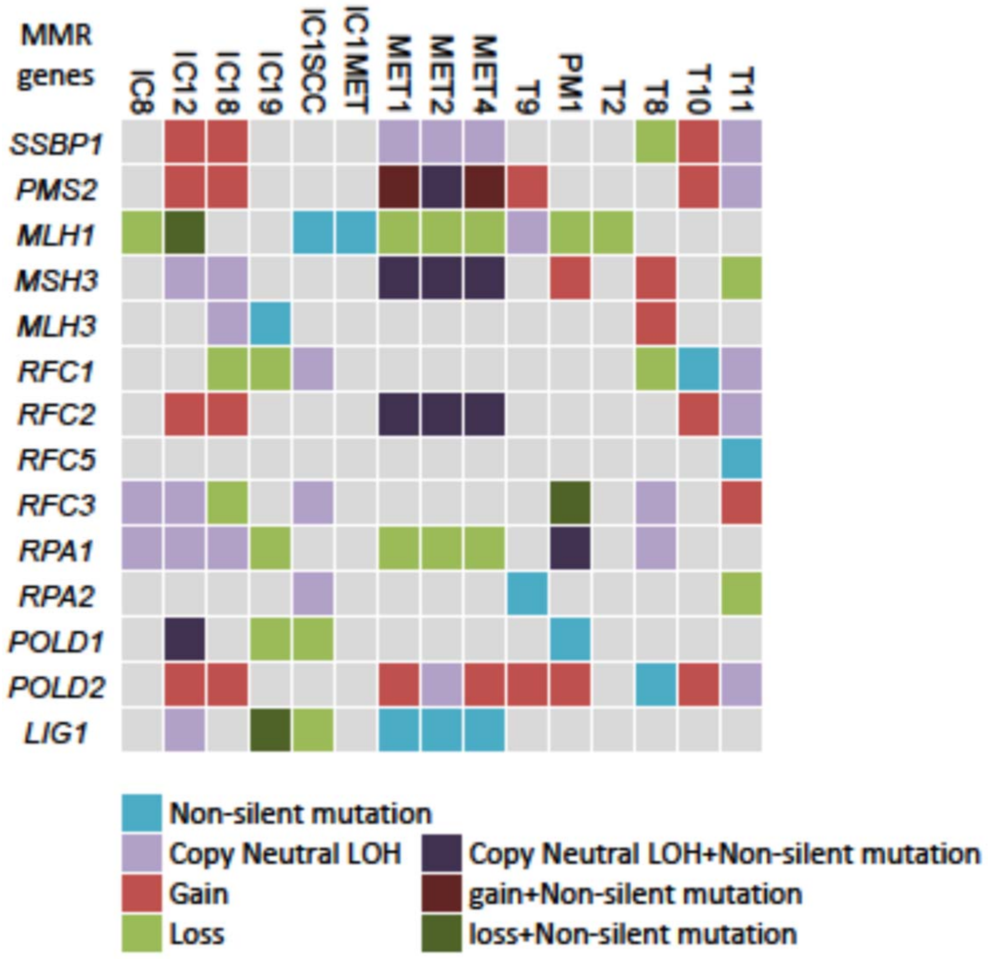

Supplementary Figure 12. OncoPrint of mismatch repair genes in cSCC cell lines

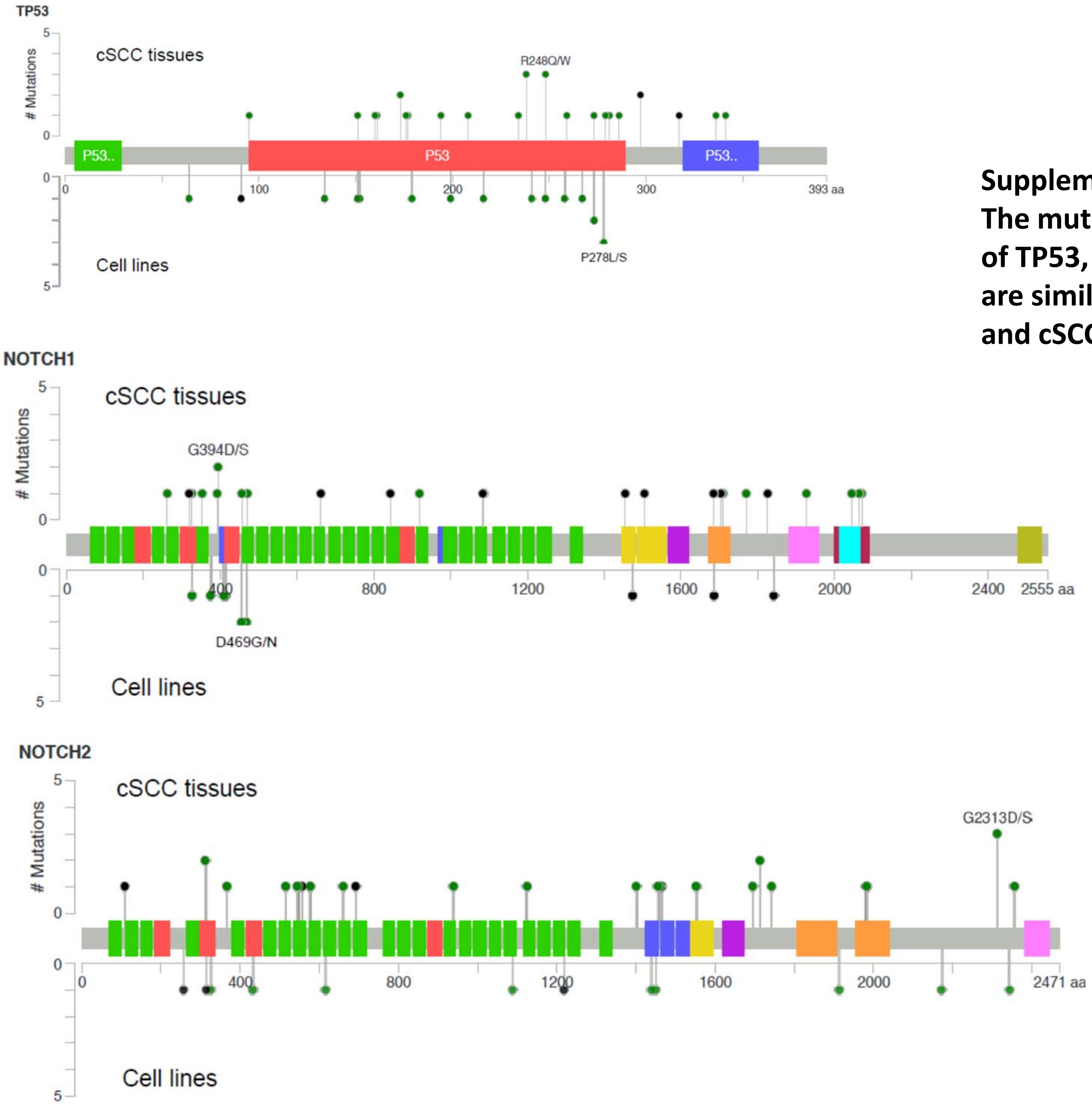

Supplementary Figure 13. The mutational profiles of TP53, NOTCH1 and NOTCH2 are similar in primary cSCC tumours and cSCC cell lines.

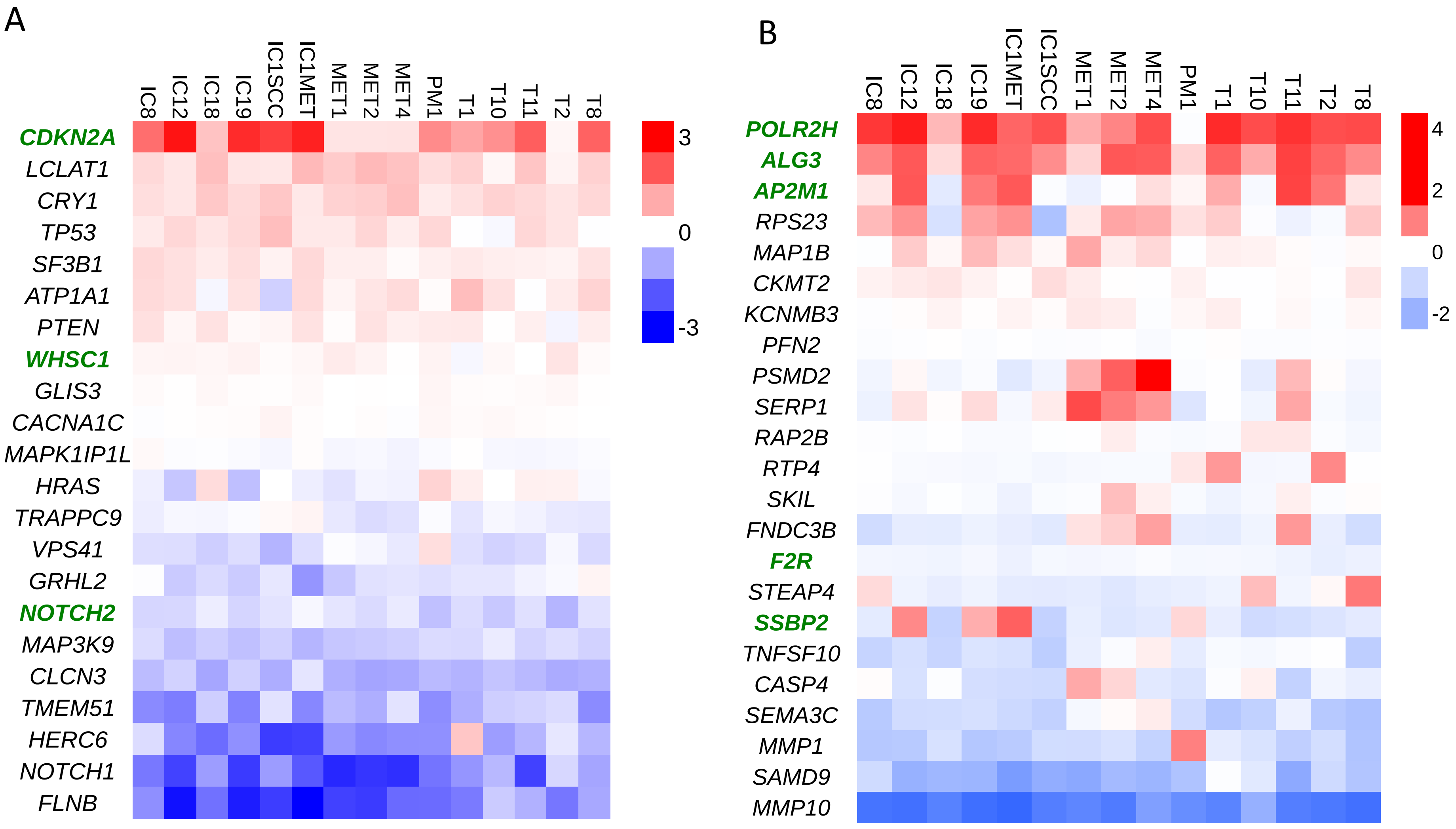

Supplementary Figure 14. Heat map analysis of gene expression changes in cSCC cell lines relative to NHKs. A) Analysis of the 22 SMGs identified in Figure 3. B) Analysis of the 23 genes of concordant chromosomal alteration identified in Figure 6. One gene NREP had no probe detected. Coincident changes in cell lines and primary tumours are highlighted in green.
