# Supplementary material for: The genomic landscape of cutaneous SCC reveals drivers and a novel azathioprine associated mutational signature
Source: Nat Commun. 2018 Sep 10;9:3667. doi: 10.1038/s41467-018-06027-1 (PMC6131170; doi:10.1038/s41467-018-06027-1)
Supplement: Supplementary file 2 — Description of Additional Supplementary Files [file 41467_2018_6027_MOESM2_ESM.pdf]

Description of Additional Supplementary Files

File Name: Supplementary Data 1
Description: 40 cSCCs - Patient/Tumour Details

File Name: Supplementary Data 2
Description: Whole exome sequencing (WES) reads yield and coverage across the 40 normal - SCC pairs

File Name: Supplementary Data 3
Description: All identified somatic mutations across 40 cSCC WES samples, with full annotation, aligned reads, VAF and clonality CCFs, subpopulation frequency estimated by EXPANDS

File Name: Supplementary Data 4
Description: Mutation signatures across all SCC exomes.

File Name: Supplementary Data 5
Description: CNAs and cn-LOH events across the 40 SCC exomes

File Name: Supplementary Data 6
Description: Significantly amplified and deleted regions across the 40 cSCC tumours based on GISTIC2.0

File Name: Supplementary Data 7
Description: Frequent copy number variations in cSCC

File Name: Supplementary Data 8
Description: Significantly mutated genes of 40 cSCC exomes based on MutSig analysis ($p < 0.05$)

File Name: Supplementary Data 9
Description: Significantly mutated genes across 40 cSCC exomes based on OncodriveFM ($q < 0.05$)

File Name: Supplementary Data 10
Description: Significantly mutated genes across 40 cSCC exomes based on OncodriveClust ($q < 0.05$)

File Name: Supplementary Data 11
Description: Somatic mutations across 40 cSCC WES samples for NOTCH1/2

File Name: Supplementary Data 12
Description: Somatic mutations across 40 cSCC WES samples for TP53 and CDKN2A

File Name: Supplementary Data 13
Description: Cross Study Comparison of Significantly Mutated Genes

File Name: Supplementary Data 14
Description: Somatic mutations across 40 cSCC WES samples for HRAS, MAP3K9, PTEN, SF3B1, VPS41 and WHSC1

File Name: Supplementary Data 15
Description: Somatic mutations across 40 cSCC WES samples for FLNB, GLIS3, CACNA1C, HERC6, TRAPPC9 and MAPK1IP1L

File Name: Supplementary Data 16
Description: Somatic mutations across 40 cSCC WES samples for GRHL2, CLCN3, TMEM51, ATP1A1, LCLAT1 and CRY1

File Name: Supplementary Data 17
Description: Comparison of 22 SMGs alteration frequencies in immunosupressed (IS) an immunocompetent (IC) patient samples

File Name: Supplementary Data 18
Description: Mutational signature analysis of mutations in 22 SMGs

File Name: Supplementary Data 19
Description: Tumour purity estimates by EXPANDS and SciClone

File Name: Supplementary Data 20
Description: Estimated number of clones in 35 Exome samples

File Name: Supplementary Data 21
Description: Rank order clonality of 22 SMGs

File Name: Supplementary Data 22
Description: Gene expression Data set 1

File Name: Supplementary Data 23
Description: Cross Study Comparison of Significantly Mutated Genes by Differentiation Status

File Name: Supplementary Data 24
Description: Kegg pathway analysis

File Name: Supplementary Data 25
Description: OncodriveFM pathway mutation significance for all profiled KEGG pathways

File Name: Supplementary Data 26
Description: cSCC Cell Line Patient Details

File Name: Supplementary Data 27
Description: All identified annotated somatic mutations in 15 cell lines

File Name: Supplementary Data 28
Description: Mutation signatures across all cSCC cell lines.

File Name: Supplementary Data 29
Description: Somatic nonsynonymous mutations in the 22 SMGs across cell lines

File Name: Supplementary Data 30
Description: Differential expression analysis of SCC cell lines against normal human keratinocytes
